# Immune–proteo–metabolomic changes link to Aβ and tau pathology in Alzheimer disease

**DOI:** 10.1002/alz.71359

**Published:** 2026-04-14

**Authors:** Meng Wang, Maria Buthut, Jenny Meinhardt, Carolin Otto, Gerardina Gallaccio, Camila Fernández‐Zapata, Matteo Teves, Claudia Samol, Katja Dettmer, Simon Heckscher, Sakshi Kamboj, Yozlem Bahar, Christian Conrad, Christian Böttcher, Desiree Kunkel, Klemens Ruprecht, Friedemann Paul, Peter J. Oefner, Helena Radbruch, Wolfram Gronwald, Harald Prüß, Chotima Böttcher

**Affiliations:** ^1^ Experimental and Clinical Research Center a cooperation between the Max Delbrück Center for Molecular Medicine in the Helmholtz Association and Charité – Universitätsmedizin Berlin Berlin Germany; ^2^ Max Delbrück Center for Molecular Medicine in the Helmholtz Association (MDC) Berlin Germany; ^3^ Department of Neurology and Experimental Neurology Charité – Universitätsmedizin Berlin Berlin Germany; ^4^ German Center for Neurodegenerative Diseases (DZNE) Berlin Germany; ^5^ Department of Neuropathology Charité – Universitätsmedizin Berlin Berlin Germany; ^6^ III. Department of Medicine University Medical Center Hamburg‐Eppendorf Hamburg Germany; ^7^ Hamburg Center for Kidney Health (HCKH) University Medical Center Hamburg‐Eppendorf Hamburg Germany; ^8^ Institute of Functional Genomics University of Regensburg Regensburg Germany; ^9^ Max Delbrück Center for Molecular Medicine Berlin Institute for Medical Systems Biology Berlin Germany; ^10^ Center of Digital Health Berlin Institute of Health at Charité – Universitätsmedizin Berlin Berlin Germany; ^11^ Flow & Mass Cytometry Core Facility Berlin Institute of Health at Charité – Universitätsmedizin Berlin Berlin Germany; ^12^ Neuroscience Research Center Charité – Universitätsmedizin Berlin Berlin Germany

**Keywords:** Alzheimer's disease, amyloid‐beta, aryl hydrocarbon receptor, mass cytometry, tau pathology, tryptophan

## Abstract

**INTRODUCTION:**

Tryptophan metabolism is increasingly implicated in Alzheimer's disease (AD), particularly through aryl hydrocarbon receptor (AhR) ligands that influence neuroinflammation. However, their relationships with core AD pathology—amyloid‐β (A) and tau (T) deposition—and associated immune–proteomic alterations remain unclear.

**METHODS:**

We performed integrative multi‐omics/high‐dimensional profiling of cerebrospinal fluid (CSF) and peripheral blood from A‐T‐ (*n* = 19) and A+T+ (*n* = 35) individuals, classified based on CSF Aβ and pTau181 levels. Analyses included targeted metabolomics, mass cytometry, and NULISA‐based proteomics, and inter‐compartmental correlation analysis. Brain‐derived tryptophan catabolism was investigated using single‐nucleus RNA sequencing (snRNA‐seq).

**RESULTS:**

Thirteen differentially expressed CSF proteins in A+T+ individuals correlated positively with tryptophan metabolites and pyroglutamate, and negatively with regulatory T cells, isobutyrate, and dendritic cells. Similar patterns were observed in blood. snRNA‐seq suggested partial brain origin of metabolites.

**DISCUSSION:**

Our findings highlight conserved immune–metabolic–proteomic signatures in AD and implicate tryptophan metabolism as a cross‐compartmental factor relevant for biomarker and therapeutic development.

**Highlights:**

- Thirteen cerebrospinal fluid (CSF) proteins involved in metabolism and neuronal function link to Alzheimer's disease (AD) pathology
- Intergrative analysis reveals shared and compartment‐specific AD signatures
- Tryptophan‐kynurenine metabolites correlate with AD pathology
- Indole metabolites show CSF‐plasma coupling in A+T+ individuals
- Immune signatures diverge across CSF (regulatory T cells [Tregs], dendritic cells [DCs]) and blood (B and myeloid cells)

## 1 BACKGROUND

Alzheimer's disease (AD) is a progressive neurodegenerative disease characterized by the accumulation of β‐amyloid (Aβ) plaques and tau‐containing neurofibrillary tangles in the brain, leading to synaptic dysfunction, neuronal loss and cognitive decline.^1^ These protein aggregates, though hallmark features of AD, represent only part of a complex and heterogeneous disease process. Growing evidence suggests that diverse biological mechanisms beyond Aβ and tau pathology contribute to AD pathogenesis. These include compartmentalized immune dysfunction,^2^, ^3^ alteration in lipid metabolism,^4^ and disruptions in amino acid and glucose metabolism.^5^ Among these processes, metabolic disturbances have emerged as critical contributors to AD. Recent research has highlighted a complex interplay between AD and tryptophan metabolites, including those derived from both the kynurenine and indole pathways. Dysregulation of the kynurenine pathway, the primary route of tryptophan catabolism, has previously been reported in AD.^6^ One metabolite of interest is quinolinic acid (QA), a neurotoxic N‐methyl‐D‐aspartate (NMDA) receptor agonist and major product of tryptophan‐kynurenine pathway.^7^ It is believed to induce oxidative stress, potentially contributing to neurodegeneration in AD and related disorders. In parallel, the indole pathway, largely driven by gut microbiota, produces a diverse array of tryptophan‐derived metabolites, many of which, such as indole‐3‐propionic acid (IPA), and indole‐3‐lactic acid (ILA),^8^ act as endogenous and microbiota‐derived ligands of aryl hydrocarbon receptor (AhR), a ligand‐activated transcription factor and key immune modulator. In the context of AD, AhR signaling may play a dual role. While AhR activation can exert anti‐inflammatory and potentially neuroprotective effects under certain conditions,^9^ chronic activation may drive oxidative stress, neuroinflammation and cognitive deficits.^10^ Aberrant AhR activation by these metabolites may influence Aβ aggregation and tau phosphorylation in AD.^11^ Human data are scarce, but studies have reported altered levels of kynurenine pathway metabolites in the blood and cerebrospinal fluid (CSF) of AD patients.^12^, ^13^

At the proteomic level, recent high‐dimensional analysis of CSF from AD patients identified distinct protein signatures associated with disease stage.^14^ Many of these proteins are linked to inflammation, synaptic function and energy metabolism, particularly alpha‐enolase (ENO1) and gamma‐enolase (ENO2), as well as tyrosine 3‐monooxygenase/tryptophan 5‐monooxygenase activation protein theta (YWHAQ) and fatty acid binding protein 3 (FABP3), which have been connected to the AhR pathway and tau pathology.^15^, ^16^, ^17^

**RESEARCH IN CONTEXT**

1. **Systematic review**: We reviewed the literature using PubMed and recent conference abstracts to identify studies examining immune, proteomic, and metabolomic changes associated with amyloid‐β and tau pathology in Alzheimer's disease (AD). Prior research has implicated tryptophan metabolism, immune cell dysregulation, and protein biomarkers in AD, but few studies have integrated these findings across cerebrospinal fluid (CSF) and blood using a systems‐level approach.
2. **Interpretation**: Our integrative analysis reveals both conserved and compartment‐specific immune–proteo–metabolomic networks associated with amyloid and tau (AT) pathology. Tryptophan metabolites—especially from the kynurenine and indole pathways—correlated with tau pathology and cognitive performance. Immune profiles diverged across compartments, highlighting distinct local and systemic responses.
3. **Future directions**: Future studies should validate the identified proteo–metabolomic‐immune signatures in larger, longitudinal cohorts and explore the functional and mechanistic roles of tryptophan metabolism and immune subsets in AD progression, including potential therapeutic targeting of gut–brain axis.

In our previous work, we observed that myeloid cells in AD patients display compartment‐specific phenotypic and metabolic changes compared to healthy controls.^3^ Specifically, myeloid cells within the central nervous system (CNS) showed signs of altered activation and metabolism, consistent with a dysregulated or exhausted immune state. These findings suggest a potential link between metabolic dysregulation and immune cell phenotype in AD.

Building on this, the present study aimed to further dissect the association between AD pathophysiology (i.e., Aβ and tau pathology), immune phenotypes, circulating metabolites and protein expression across CSF and blood compartments. We employed a systems‐level integrative approach to analyze matched CSF and blood specimens from individuals with and without AD pathology (A‐T‐ and A+T+ according to ATN‐classification^18^). Using targeted proteomics via the NULISA platform, metabolomics and mass cytometry (CyTOF) for in‐depth immune profiling, we identified significant alterations in CSF protein profiles between A‐T‐ and A+T+ individuals. These differences were enriched in pathways related to metabolism, immune response and synaptic function. Through integrative analysis using the DIABLO framework, we uncovered key molecular features associated with AD pathology across multiple data layers. Core proteins, including phosphorylated Tau (pTau), MAPT, FABP3, MIF and NRGN, were linked to the kynurenine and indole pathways, as well as broader amino acid and energy metabolism. These proteins also showed associations with compartment‐specific immune populations, particularly regulatory T cells and CD1c^+^CD49d^+^ myeloid cells in CSF. Together, our findings highlight cellular and molecular interactions in AD and may inform future strategies to prevent or intervene in the progression of AD pathology.^19^

## 2 METHODS

### 2.1 Study participants and clinical data

To better understand compartment‐specific differences in immune and molecular networks underlying AD pathology, we collected paired CSF‐blood specimens from A+T+ (*n* = 35) and A‐T‐ (*n* = 19) patients. Specimens were collected as part of routine diagnostics at the neurological memory clinic at Charité Campus Mitte, Universitätsmedizin Berlin. This study was conducted according to the Declaration of Helsinki and its later amendments. All patients were categorized according to the ATN framework for clinical documentation. However, in our study, only A and T status were used to define study groups: A+T+ individuals were classified as having biomarker‐defined AD, whereas A‐T‐ individuals were classified as non‐AD. For defining the Aβ and tau pathologies, reduced CSF amyloid‐β42 concentrations or positive amyloid positron emission tomography (PET) scans indicate the presence of amyloid pathology (A), while elevated CSF phosphorylated tau (primarily p‐tau181) or tau PET imaging reflects the presence of insoluble tau fibrils (T).^18^ Cognitive measures (Montreal Cognitive Assessment [MoCA] and Mini‐Mental State Examination [MMSE]) were recorded to describe clinical characteristics of the cohort but were not used for group assignment. In this cohort, patients with MoCA scores of 20‐25 or MMSE scores of 23‐27 corresponded to mild cognitive impairment (MCI), Whereas patients with MoCA scores of 15–20 and MMSE scores < 22 in a mixed‐age cohort corresponded to dementia.^20^, ^21^ The demographic and clinical characteristics of each patient are summarized in Table S1.

### 2.2 Sample collection for mass cytometry

All blood and CSF specimens were maintained at 4°C during processing and prepared within 1 h of collection. Five hundred µL of ethylenediaminetetraacetic acid (EDTA) whole blood (WB) were immediately fixed with 700 µL of Proteomic Stabilizer (Smart Tube Inc.) according to manufacturer specifications and stored at 80°C until CyTOF analysis. CSF samples were centrifuged (300 × g, 10 minutes, 4°C) to pellet cells, which were resuspended with 10% bovine serum albumin (BSA) and Smart Tube Stabilizer and stored at 80°C until CyTOF analysis.

### 2.3 CyTOF experiment

Cells were analyzed using a CyTOF2 upgraded to Helios specifications, using a narrow bore injector. It is important to note that only CSF samples containing < 100 erythrocytes/µL were included. CSF and WB samples underwent separate staining procedures to maintain protocol specificity for each sample type.

### 2.4 Intracellular barcoding for CyTOF

Samples were processed according to a standardized barcoding workflow. After thawing in Thaw/Lyse buffer, samples were labeled with unique combinations of palladium isotopes (^102^Pd, ^104^Pd, ^105^Pd, ^106^Pd, ^108^Pd, and ^110^Pd) using the Cell‐ID 20‐plex Pd Barcoding Kit (StandardBio). This combinatorial approach assigns three of the six isotopes per sample, enabling the generation of 20 distinct barcode combinations. The barcoding incubation was carried out at room temperature for 30 minutes, after which individual samples were washed twice with staining buffer (phosphate buffered saline [PBS] containing 0.5% bovine serum albumin [BSA] and 2 mM EDTA). Finally, all samples were pooled, washed again, and prepared for subsequent antibody staining procedures.

### 2.5 Antibodies

Antibodies were acquired either pre‐conjugated to metal isotopes (Standard BioTools) or in purified form commercial vendors, with unconjugated antibodies conjugated in‐house using the MaxPar X8 kit (Standard BioTools) per manufacturer's protocol. Two antibody panels containing 37 antibodies each were papered and restored at ‐80°C until CyTOF staining: Panel A was designed for major B cell and other myeloid and T cell subsets characterization, while antibody panel B targets circulating immune cells and their subsets, including T cells and myeloid cells (Table S2 for Panel A, Table S3 for Panel B). All antibodies were validated for use in human immune cells using CyTOF.

### 2.6 Surface and intracellular staining

After sample pooling, barcoded cells were first incubated with Human Fc receptor blocking solution (BioLegend; 1:160 dilution in staining buffer) for 10 min at 4°C to minimize nonspecific binding. Surface marker staining was then performed by adding 90 µL of surface antibody cocktail and incubating for 30 min at 4°C. After two washes with staining buffer, cells were fixed overnight in 2% methanol‐free formaldehyde (FA) solution at 4°C. For intracellular staining, fixed cells were washed once with staining buffer before incubation with 100 µL of intracellular antibody cocktail in permeabilization buffer (30 minutes, room temperature). Following two additional washes, cells were incubated with 1 mL iridium intercalator solution (Fluidigm; 125 µM stock; diluted 1:1000 in 2% FA, final concentration 0.125 µM) for 30 minutes at room temperature to enable cell identification during mass cytometry. Prior to CyTOF acquisition, cells were maintained at 4°C in staining buffer and subjected to a final wash procedure using a laminar flow system (Mini‐1000, Curiox Biosystems) with MilliQ water. To control for technical variability, we incorporated anchor samples derived from healthy donor WB processed identically to patient samples in all experimental batches.

### 2.7 CyTOF data analysis

As described before,^3^, ^22^ nucleated single intact cells were manually gated according to the signals of DNA intercalators ^191^Ir/^193^Ir and event length. Samples were de‐barcoded based on unique palladium barcode combinations (^10^ ^2^Pd/^10^ ^4^Pd/^10^ ^5^Pd/^10^ ^6^Pd/^10^ ^8^Pd/^1^ ^10^Pd) using FlowJo software. Following de‐barcoding, all samples were exported as individual FCS files. Each FCS file was cleaned and compensated for signal spillover using R package *CATALYST*,^23^ transformed with arcsinh transformation (scale factor 5), and batch correction was implemented with a quantile normalization method to minimize batch effects.^24^ Prior to clustering analysis, CSF and WB cells were pre‐gated. The pre‐gating strategy can be viewed in Figure S1(A) (for CSF) and Figure S2 (for WB). CSF cells were pre‐gated as follows: for Panel A and Panel B, CD3^+^CD19^−^ T cells, along with CD3^−^CD19^−^ myeloid and NK (MNK) cells were extracted. For WB samples, in Panel A, CD3^−^CD19^+^ B cells were selected. In panel B, cPARP^−^CD3^+^ T cells and cPARP^−^CD3^−^CD66b^−^CD14^−/+^ MNK cells were pre‐gated using FlowJo. For downstream analysis, we used previously described workflows.^25^ Only CSF samples with > 10 cells were considered for the downstream data analysis. For unsupervised cell population identification, we performed cell clustering with the *FlowSOM* and *ConsensusClusterPlus* algorithms using selected markers in each panel (Table S4 for CSF, Table S5 for WB). These selected markers were identified by the principal component analysis (PCA) ‐based non‐redundancy score (NRS),^25^ which is suggestive of markers that explain most of the variability among samples. We then chose the twelve highest scoring NRS markers plus other lineage markers for clustering. The number of meta‐clusters used for further analysis was identified based on the delta area plots^25^ (which assess the “natural” number of clusters that best fits the complexity of the data) together with visual inspection of the phenotypic heatmap with the aim of selecting a cluster number with consistent phenotypes that would also allow exploration of small populations. Cell population characterization was performed through two visualization approaches: (1) Cluster heatmaps displaying the scaled median marker expression intensities, where population phenotypes (marker‐positive vs. marker‐negative) were determined by relative expression compared to other clusters, and (2) uniform manifold approximation and projection (UMAP) with cluster overlays, generated using selected markers as input and normalized through down‐sampling (maximum 1000 cells/sample) to ensure balanced representation (Figure S1(B) for CSF, Figure S3 for WB).

### 2.8 NULISAseq assay

The concentrations of CSF proteins were measured using NULISAseq assays, which were performed at Alamar Biosciences, USA.^26^ These proteins mainly target neurodegenerative disease‐related protein markers and immune response‐related cytokines/chemokines. The Alamar NULISA immunoassay utilizes matched antibody pairs for each target, consisting of (1) a capture antibody conjugated to a partially double‐stranded DNA molecule with a poly‐A tail (for oligo‐dT bead immobilization) and a target‐specific molecular identifier, and (2) a detection antibody conjugated to a complementary DNA strand containing a biotin group (for streptavidin bead capture) and matching target‐specific barcode. Following target binding, the proximal DNA strands are ligated to form a sequencer‐compatible template, with subsequent quantification achieved through next‐generation sequencing of the barcoded reporters, where target abundance correlates directly with barcode read counts. Protein concentrations are normalized using internal and inter‐plate controls, then log_2_‐transformed to generate NULISA Protein Quantification (NPQ) units for downstream analyses.

### 2.9 Nuclear magnetic resonance spectroscopy

Metabolite and lipoprotein profiling of plasma specimens was performed using standardized, fully automated nuclear magnetic resonance (NMR) protocols from Bruker BioSpin GmbH (Rheinstetten, Germany) for quantification of up to 41 metabolites and lipoprotein subclass analysis. For sample preparation, 286.72 µL of EDTA plasma were combined with an equal volume of Bruker plasma IVDr NMR buffer using the liquid handling robot SamplePro (Bruker). NMR analysis was conducted on a 600 MHz Bruker Avance III spectrometer equipped with a cryogenic triple‐resonance probe (^1^H/^1^ ^3^C/^1^ ^5^N with ^2^H lock), z‐gradients, and an automated temperature‐controlled sample handler. Four complementary NMR experiments were acquired at 310 K: 1D ^1^H NOESY, 2D ^1^H JRES, 1D ^1^H CPMG, and 1D ^1^H diffusion experiments, following Bruker IVDr specifications. Acquired data were automatically processed through the Bruker analysis platform, providing absolute free concentrations of up to 41 metabolites and 134 lipid parameters for downstream analysis.

CSF samples (300 µL) were prepared for NMR analysis by mixing with an equal volume of NMR buffer (Bruker BioSpin) and 20 µL of 240 mmol/L formic acid (FA) as a protein‐binding‐resistant internal standard. Using the same 600 MHz Bruker Avance III spectrometer described previously, each sample underwent 1D Carr‐Purcell‐Meiboom‐Gill (CPMG) experiments at 298 K. Data processing was performed semi‐automatically using TopSpin 4.0.7 software (Bruker BioSpin), followed by spectral quantification with Chenomx 8.6 (Chenomx Inc.), which enabled precise measurement of 25 metabolites per sample.

### 2.10 Liquid chromatography‐tandem mass spectrometry of tryptophan metabolites

Tryptophan and its key metabolites were quantified in plasma and CSF samples as described previously, if not stated otherwise.^27^ Shortly, 10 µL of an aqueous stable isotope labeled internal standard mixture containing ^13^C_6_‐anthranilic acid, ^13^C_7_‐3‐hydroxyanthranilic acid, ^2^H_5_‐hydroxyindoleacetic acid, ^13^C_2_‐indole‐3‐acetic acid, ^13^C_10_‐kynurenic acid ^13^C_10_‐kynurenine, ^2^H_4_‐nicotinamid, ^2^H_4_‐nicotinic acid, ^2^H_4_‐serotonine, ^2^H_4_‐tryptamine, ^2^H_3_‐L‐DOPA, ^13^C_2_‐^15^N_1_‐3‐hydroxykynurenine, ^2^H_4_‐xanthurenic acid, ^2^H_2_‐3‐indolepropionic acid and ^2^H‐indolelactic acid (10 µM each), as well as ^13^C_11_‐tryptophan (200 µM) and ^2^H_3_‐quinolinic acid (100 µM) was added to up to 50 µL of plasma or 50–100 µL CSF. Then, the sample was diluted in a ratio 1:5 with cold 100% MeOH, vortexed, and incubated at −80°C for 24 hours to ensure complete protein precipitation. Samples were centrifuged at 10,000×g at 4°C for 5 minutes, the supernatant was collected. The protein pellets were washed twice with 200 µL of cold 80% MeOH. After centrifugation at 10,000×g at 4°C for 5 minutes both supernatants of the washing steps were combined with the first supernatant. The collected supernatants were dried using a vacuum evaporator (CombiDancer, Hettich AG, Bach, Switzerland). The residue was redissolved in 100 µL of 0.1% formic acid in water. Chromatographic separation of metabolites was achieved using an ACQUITY Premier HSS T3, 1.8 µm, 2.1 × 150 mm column (Waters, Germany, Eschborn). Using an ExionLC‐30AD HPLC system (AB Sciex, Germany, Darmstadt), gradient elution was carried out mobile phase A consisting of 0.1% formic acid in water and mobile phase B of 0.1% formic acid in acetonitrile. Metabolite detection was performed with a 6500^+^ Triple Quadruple mass spectrometer (AB Sciex, Germany, Darmstadt). Peak integration and data evaluation were done using SciexOS‐MQ Software (Version 242 2.1.6, AB Sciex, Germany, Darmstadt).

### 2.11 Integrative analysis

To identify cross‐dataset signatures, we employed Data Integration Analysis for Biomarker Discovery using Latent Components (DIABLO) from the mixOmics package. This multi‐block sparse partial least squares discriminant analysis (sPLS‐DA) method performs supervised integration by maximizing covariance between paired datasets while maintaining discriminative power for the outcome variable. Our analysis used a weighted design matrix (between‐dataset weight = 0.1; dataset‐outcome weight = 1) to optimize biological pattern discovery over technical integration. To reduce computational time, maximum 15 differential variables were pre‐selected from each dataset; note that we only specified the number of variables to retain, while the model itself identified the individual variables.

Variable selection in DIABLO is based on a penalized regression framework that balances two objectives: maximizing correlation (covariance) across datasets and optimizing discrimination of the outcome variable. For each dataset, latent components are constructed as linear combinations of variables, and the model applies L1‐penalization to induce sparsity, selecting only those variables that contribute most to both shared variation across omics layers and class separation. Thus, the selected variables represent a minimal and correlated subset that jointly captures multi‐modal biological signals relevant to the phenotype of interest. Results were visualized in a reduced dimensional space using the first two weighted principal components.

### 2.12 Single‐nucleus RNA‐sequencing analysis

The snRNA‐sequencing (snRNA) dataset was available from a previously published study.^28^ Briefly, the data were derived from post‐mortem human brainstem samples (*n* = 12; *n* = 4 AD, *n* = 8 non‐demented individuals, such as coronavirus disease 2019 [COVID‐19], amyotrophic lateral sclerosis [ALS], and others). In this dataset, compared to microglia/macrophage (MG/Mac) cluster 2, MG/Mac cluster 1 exhibits lower expression of *PTPRC* (CD45), *CD47*, *ITGAM* (CD11b), *CD14*, and *CD68* genes, while showing higher expression of *P2RY12* and *TMEM119* homeostatic markers. Among endothelial cell populations, cluster 1 corresponds to venous endothelial cells, cluster 2 to arterial endothelial cells, and cluster 3 to capillary endothelial cells. Neuronal clusters do not display exclusive neurotransmitter expression profiles.

### 2.13 Statistical analysis

All analyses and plots were performed in R v.4.2.2 or GraphPad Prism v.10.2.0. Continuous variables (age and clinical characteristics) were compared between the A‐T‐ and A+T+ groups using the Mann–Whitney U test, while categorical variables (gender distribution) were analyzed using the Pearson's Chi‐Square Test. Initial comparisons revealed no significant differences in age or gender distribution between A‐T‐ and A+T+ groups across all data layers. Consequently, these demographic variables were excluded as covariates in subsequent analyses. Differential protein expression between A‐T‐ and A+T+ groups was assessed using linear mixed‐effects models implemented in the limma R package (v.3.58.1). To control for multiple comparisons, we applied Benjamini–Hochberg false discovery rate (FDR) correction, with statistically significant differentially expressed proteins (DEPs) defined by an FDR‐adjusted *p*‐value threshold of 0.05. For comparison of other omics data (metabolites, lipoproteins, and proportion of immune cells), the Mann–Whitney U test (two‐tailed, unpaired) was used. To control for multiple testing, *p*‐values were adjusted using the Two‐stage Step‐up procedure of Benjamini, Krieger, and Yekutieli (BKY) to control the FDR at 5%. Differential gene expression test was performed using the function FindMarkers from the R package Seurat (v.5.3.0) with the MAST algorithm. Genes were considered significantly differentially expressed when they satisfied the criteria of log2(fold change) > 0.25 and *p* value < 0.05. *p*‐Values were adjusted using the Benjamini–Hochberg procedure, and significance was defined as an FDR‐adjusted *p* < 0.05. Cross‐layers relationships between CSF NULISA DEPs and other molecular profiles (metabolites, lipoproteins, immune cells) were assessed using DIABLO integration. The statistical significance was set at a correlation coefficient of |*r*| ≥ 0.6. Significant differences in concentration of tryptophan metabolites between CSF and plasma in both A‐T‐ and A+T+ groups were calculated using Wilcoxon matched‐pairs signed rank test. Correlations between tryptophan metabolites across compartments in the A‐T‐ and A+T+ groups were analyzed using Spearman correlation.

## 3 RESULTS

### 3.1 Study design and clinical parameters

**FIGURE 1:**
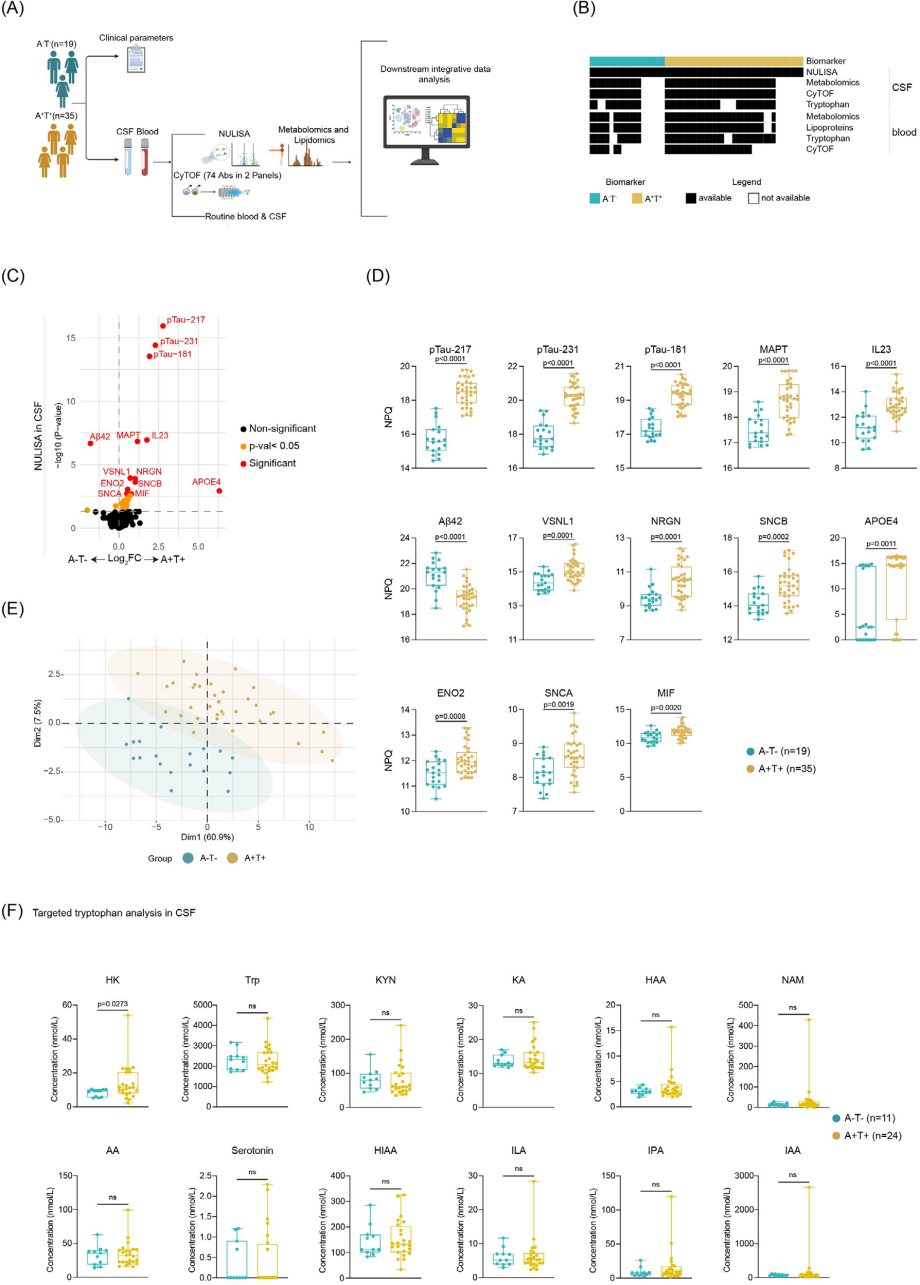
Study overview and CSF proteomic differences by AT classification. (A) Schematic overview of the study workflow. Individuals were stratified into A+T+ (*n* = 35) and A‐T‐ (*n* = 19) groups based on established CSF biomarkers of AD pathology. CSF and whole blood specimens were collected for integrative analyses. Immune cell populations in CSF and WB were profiled using CyTOF with two antibody panels. Remaining CSF and plasma samples were subjected to targeted metabolomics, NMR‐based lipoprotein subclass analysis, and proteomic analysis using the NULISA platform, alongside routine clinical laboratory tests. All datasets were integrated for downstream analysis. (B) Sample overview illustrating data availability across individuals and omics platforms, stratified by AT category. (C) Volcano plot showing DEPs between A‐T‐ (*n* = 19) and A+T+ (*n* = 35) groups. Protein abundance was compared using a linear mixed‐effects model. Significance was determined using the Benjamini–Hochberg method to correct for multiple testing. Proteins with an adjusted *p*  <  0.05 are highlighted in red, nominal *p*  <  0.05 in blue, and non‐significant proteins in black. Each dot represents a single protein; the horizontal dashed line indicates the unadjusted *p*  =  0.05 threshold. (D) Box plots showing NPQ values for DEPs between A‐T‐ and A+T+ individuals from NULISA experiment. Each dot represents an individual patient. (E) PCA of NULISA‐derived CSF proteomic data from A‐T‐ and A+T+ individuals. Each point represents one sample projected onto the first two principal components (Dim1 and Dim2). (F) Box plots illustrating the concentrations of significantly different tryptophan metabolites in CSF between A‐T‐ and A+T+ groups. Statistical significance was determined using the Mann–Whitney U test (two‐tailed, unpaired). To control for multiple testing, *p*‐values were adjusted using the two‐stage step‐up procedure of Benjamini, Krieger, and Yekutieli to control the false discovery rate at 5%. AD, Alzheimer's disease; AT, Aβ and tau; CyTOF, mass cytometry; CSF, cerebrospinal fluid; DEPs, differentially expressed proteins; NMR, nuclear magnetic resonance; NULISA, nucleic acid linked immuno‐sandwich assay; NPQ, NULISA protein quantification; PCA, principal component analysis; WB, whole blood.

To explore the molecular and cellular features associated with AD pathology, we performed integrative analyses on paired CSF and WB samples from individuals classified as A‐T‐ (Aβ and tau negative, *n* = 19) and A+T+ (Aβ and tau positive, *n* = 35) based on established AD biomarkers (see Methods). Clinical information, including disease duration and comorbidity frequencies, as well as between‐group comparisons, is shown in Figure S4. CSF profiling included nucleic acid linked immuno‐sandwich assay (NULISA)‐based targeted proteomics, targeted metabolomics, and immune cell phenotyping by CyTOF. In parallel, WB samples and EDTA plasma were analyzed for immune cell populations and plasma metabolites and lipoprotein (sub‐)classes (Figure 1(A),(B)). Despite the difference in sample size for each omics layer, no significant difference was observed in the distribution of sex and age between the two groups (Table 1). The clinical data analysis of the CSF revealed that the A+T+ group exhibited significantly higher levels of t‐Tau and pTau‐181, whereas the A‐T‐ group showed elevated Aβ42 and Aβ42/40 ratios. In contrast, Aβ40 and neurofilament‐light (NfL) levels did not differ significantly between groups (Figure S5).

**TABLE 1 alz71359-tbl-0001:** Clinical and demographic characteristics of participants

Characteristics	A‐T‐	A+T+	*p*‐Value
**CSF dataset**			
NULISA (*n*)	19	35	
Age mean (SD), y	71.8 (8.3)	70.9 (7.6)	0.6903^a^
Gender (female/male)	7/12	13/22	0.9826^b^
Metabolomics (*n*)	13	28	
Age mean (SD), y	70.8 (8.5)	72.0 (6.0)	0.6178^a^
Gender (female/male)	4/9	9/19	0.9299^b^
CyTOF_CD3negA (*n*)	13	27	
Age mean (SD), y	70.8 (8.5)	72.2 (6.0)	0.5673^a^
Gender (female/male)	4/9	9/18	0.8712^b^
CyTOF_CD3posA (*n*)	13	26	
Age mean (SD), y	70.8 (8.5)	71.8 (5.8)	0.6680^a^
Gender (female/male)	4/9	8/18	>0.9999^b^
CyTOF_CD3negB (*n*)	13	24	
Age mean (SD), y	70.8 (8.5)	71.2 (5.9)	0.8937^a^
Gender (female/male)	4/9	6/18	0.7060^b^
CyTOF_CD3posB (*n*)	11	21	
Age mean (SD), y	69.8 (8.9)	72.0 (5.3)	0.3901^a^
Gender (female/male)	4/7	5/16	0.4531^b^
Tryptophan (*n*)	11	24	
Age mean (SD), y	70.3 (8.4)	71.6 (6.2)	0.5978^a^
Gender (female/male)	4/7	7/17	0.6703^b^
**Whole blood dataset**			
Metabolomics (*n*)	12	26	
Age mean (SD), y	70.6 (8.8)	72.5 (5.8)	0.4199^a^
Gender (female/male)	3/9	9/17	0.5534^b^
Lipidomics (*n*)	12	26	
Age mean (SD), y	70.6 (8.8)	72.5 (5.8)	0.4199^a^
Gender (female/male)	3/9	9/17	0.5534^b^
Tryptophan (*n*)	11	26	
Age mean (SD), y	71.5 (8.7)	72.5 (5.9)	0.6718^a^
Gender (female/male)	3/8	8/18	0.8316^b^
CyTOF (*n*)	7	22	
Age mean (SD), y	71.0 (9.7)	72.5 (5.7)	0.6256^a^
Gender (female/male)	2/5	9/13	0.5579^b^
**Clinical parameter (CSF)**			
NfL (pg/mL) (*n*)	11	23	
mean (SD)	1212.4 (707.9)	1724 (1219)	0.1532^a^
Aβ40 (pg/mL) (*n*)	19	33	
mean (SD)	6892.5 (2644.4)	8053.9 (3235.6)	0.2816^a^
Aβ42 (pg/mL) (*n*)	19	32	
mean (SD)	904.4 (350.9)	405.0 (137.7)	<0.0001^a^
Aβ42/40 ratio (*n*)	19	34	
mean (SD)	0.13 (0.03)	0.05 (0.02)	<0.0001^a^
tTau (pg/mL) (*n*)	19	34	
mean (SD)	395.6 (111.1)	794.4 (247.1)	<0.0001^a^
pTau181 (pg/mL) (*n*)	19	34	
mean (SD)	50.4 (15.5)	144.9 (32.3)	<0.0001^a^

Abbreviations: A, Aβ; Aβ, β‐amyloid; CSF, cerebrospinal fluid; CyTOF, mass cytometry; NfL, neurofilament‐light; NULISA, nucleic acid linked immuno‐sandwich assay; pTau, phosphorylated Tau; SD, standard deviation; T, tau; tTau, total Tau.

^a^ Mann–Whitney U test.

^b^ Pearson's chi‐squared test.

### 3.2 Differential analysis of CSF proteins revealed key changes associated with AD pathology

We first performed a differential analysis of soluble proteins between individuals with (A+T+) and without (A‐T‐) AD pathology. Among the 322 CSF proteins quantified by the NULISA platform,^26^ 13 differentially expressed proteins (DEPs) were identified (Figure 1(C,D)). Of these, 12 DEPs, including pTau‐217, pTau‐231, pTau‐181, microtubule‐associated protein Tau (MAPT), and IL23, were elevated in the CSF of A+T+ individuals. In contrast, Aβ42 levels were higher in A‐T‐ individuals, consistent with increased cerebral Aβ deposition in A+T+ patients^29^ (Figure 1(D)). PCA based on the DEPs (*p* < 0.05) showed a trend toward separating A‐T‐ from A+T+ individuals (Figure 1(E)). Despite these proteomic differences, high‐dimensional immune profiling using CyTOF did not reveal significant alterations in CSF immune cell composition between A‐T‐ and A+T+ groups (Figure S6), consistent with our previous observation in AD patients versus healthy individuals.^3^ Metabolomic analysis of CSF, with a focus on tryptophan‐derived metabolites, including AhR ligands, using LC‐MS/MS and NMR, revealed a significantly increased level of 3‐hydroxykynurenine (HK) in A+T+ patients (Figure 1(F)), implicating altered kynurenine pathway activity in AD pathology.

### 3.3 Cross‐high‐dimensional correlation analysis reveals cellular and molecular features in CSF associated with AD pathology

**FIGURE 2:**
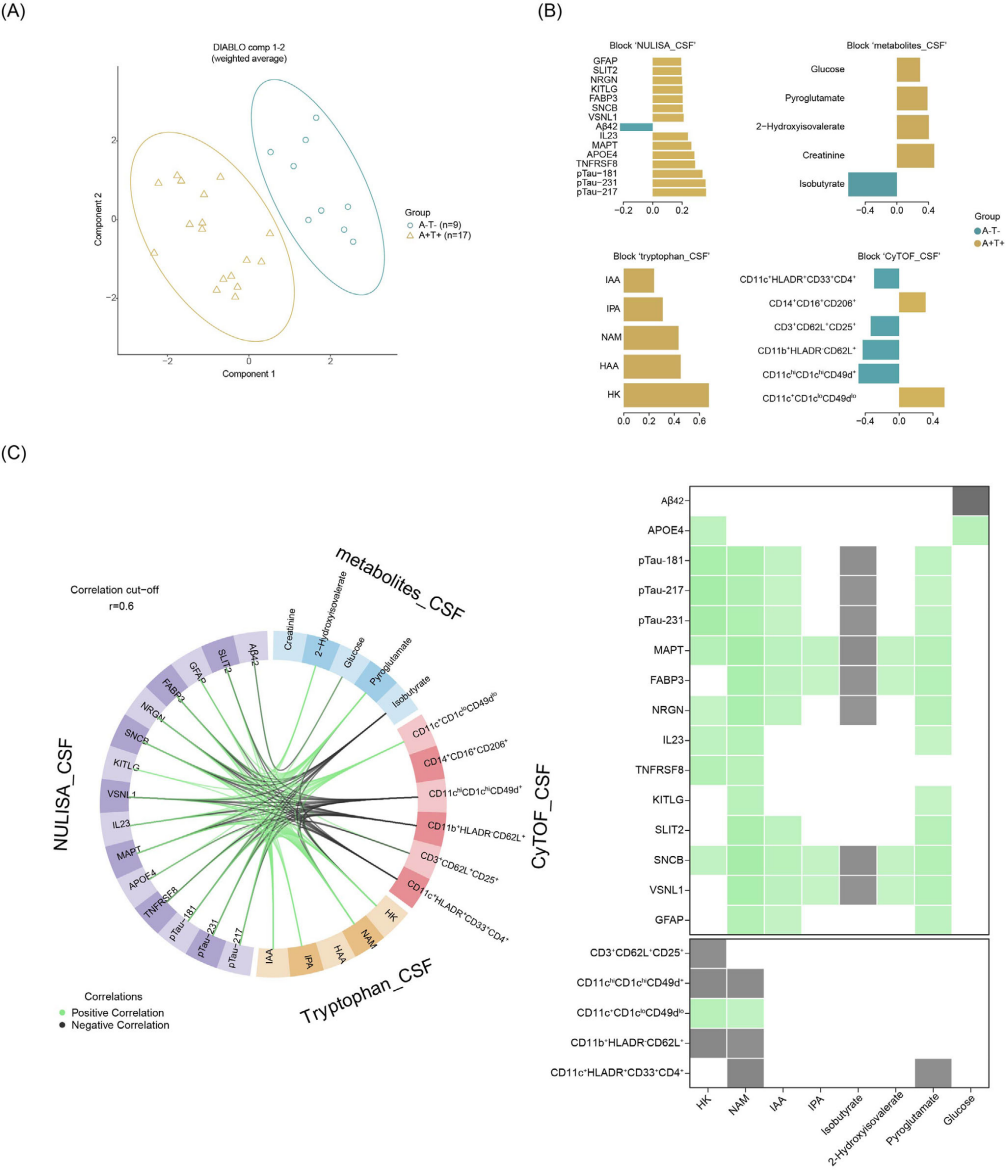
Multiple dataset integration in CSF identifies distinct molecular signatures differentiating A‐T‐ and A+T+ groups. (A) Sample distribution in the two‐component space derived from supervised discriminant analysis using multiblock partial least squares (block PLSDA/DIABLO). (B) Loading plot of variables selected on Component 1. Features are ranked from bottom to top by decreasing absolute coefficient values and are colored according to the group (A‐T‐ or A+T+) with the higher median expression. (C) Circos plot displaying interaction between variables across omics blocks with absolute correlation coefficients ≥ 0.6. Green links represent positive correlations; black links represent negative correlations. Variables are positioned by block origin and colored accordingly. The right panel summarizes these correlations in matrix format, corresponding to the interactions shown in the Circos plot. A, amyloid‐β; CSF, cerebrospinal fluid; T, tau.

To gain a systems‐level understanding of cellular and molecular correlates of AD pathology, we applied DIABLO (Data Integration Analysis for Biomarker Discovery using Latent variable approaches for Omics studies, implemented in the R package mixOmics)^30^ to paired CSF datasets including CyTOF (immune phenotypes), NULISA (proteomics), and metabolomics. DIABLO maximizes inter‐dataset correlation to identify key features discriminating A+T+ from A‐T‐ individuals, using multivariate projection‐based feature selection rather than univariate *p*‐values.^30^ Sparse partial least squares discriminant analysis (sPLS‐DA) revealed a clear separation of A+T+ and A‐T‐ individuals based on combined immune, metabolic, and proteomic profiles (Figure 2(A)). This separation was driven by 15 proteins, five classical metabolites, five tryptophan metabolites and six immune[Table alz71359-tbl-0001] cell subsets (Figure 2(B)). Most proteins overlapped with previously identified DEPs.

Beyond proteomic markers, A+T+ patients showed elevated levels of metabolites involved in energy mechanism (e.g., glucose, creatinine, pyroglutamate), branched‐chain amino acid (BCAA) metabolism (e.g., 2‐hydroxyisovalerate [2‐HIV] also known as 2‐hydroxy‐3‐methylbutyric acid, which is formed by reduction of 2‐ketoisovaleric acid, the product of oxidative deamination of valine) and inflammation (e.g., indole‐3‐acetic acid [IAA], indole‐3‐propionic acid [IPA], nicotinamide [NAM], 3‐hydroxyanthranilic acid [HAA] and HK).^11^, ^31^ Correlation analysis revealed no significant associations between age and any of the differentially expressed metabolites (Figure S7), indicating that the observed group differences are unlikely to be age‐related. In addition, A+T+ individuals had an increased proportion of CD206^+^CD14^+^CD16^+^ intermediate monocytes and CD11c^+^CD1c^lo^CD49d^lo^ myeloid cells. In contrast, A‐T‐ individuals exhibited higher levels of isobutyrate, a BCAA‐derived short‐chain fatty acid (SCFA),^32^ and a higher level of one regulatory T cell (Treg) population (CD3^+^CD25^+^CD62L^+^)^33^ along with three distinct myeloid cell subsets (Figure 2(B), Figure S1(B) for immune cell phenotype).

Notably, among the 15 discriminatory proteins identified in our study, several aligned with co‐expression modules previously described in a large‐scale AD proteomic dataset.^14^ These include proteins from the immune‐response module (e.g., KITLG, TNFRSF8) and the metabolic/tau module (e.g., MAPT, FABP3), both of which have been implicated in AD progression and cognitive decline. To explore potential crosstalk between protein, metabolite and immune features, we conducted correlation analysis using the DIABLO framework. This revealed multiple associations between discriminatory proteins, metabolites and immune cell subsets (correlation threshold *r* ≥ 0.6; Figure 2(C), left). Among all discriminating metabolites, only the kynurenine pathway metabolites HK and NAM showed positive correlations with DEPs and CD11c^+^CD1c^lo^CD49d^lo^ myeloid cells. In contrast, these metabolites negatively correlated with CD11c^hi^CD1c^hi^CD49d^+^ dendritic cells (DCs), CD11b^+^HLA‐DR^−^CD62L^+^ myeloid cells and CD3^+^CD25^+^CD62L^+^ Treg cells, all of which were more abundant in the CSF of A‐T‐ individuals (Figure 2(B),(C)). Isobutyrate correlated only with discriminatory proteins but not with immune cell subsets. At the protein level, HK and NAM were positively correlated with metabolic proteins (MAPT and/or FABP3), immune‐related proteins (IL23, TNFRSF8), tau pathology markers, and neuronal/synaptic proteins. In contrast, the indole metabolites IAA and IPA were associated mainly with metabolic proteins (MAPT, FABP3) and neuronal markers, but not with immune response proteins. Isobutyrate negatively correlated with tau pathology, metabolic and neuronal proteins, while pyroglutamate and 2‐HIV showed positive association with these same protein groups. Additionally, glucose levels were positively correlated with apolipoprotein E4 (APOE4), but negatively with Aβ42.

Collectively, our integrative CSF analysis delineates potential group‐specific molecular networks that may connect protein modules associated with tau pathology, immune activation, metabolism, and Aβ burden to distinct immune–metabolic profiles. These profiles are characterized by imbalances in myeloid and Treg cell populations, as well as pathway‐specific metabolic signatures (e.g., kynurenine and indole pathways, energy metabolism). These selective cross‐omics interaction distinguish A+T+ from A‐T‐ individuals and underscore the compartmentalized immune–metabolic crosstalk of AD pathology. Nevertheless, translating these correlative findings into functional insights remains technically challenging in human systems.

### 3.4 Cross‐compartment dysregulation of tryptophan metabolism in AD

**FIGURE 3:**
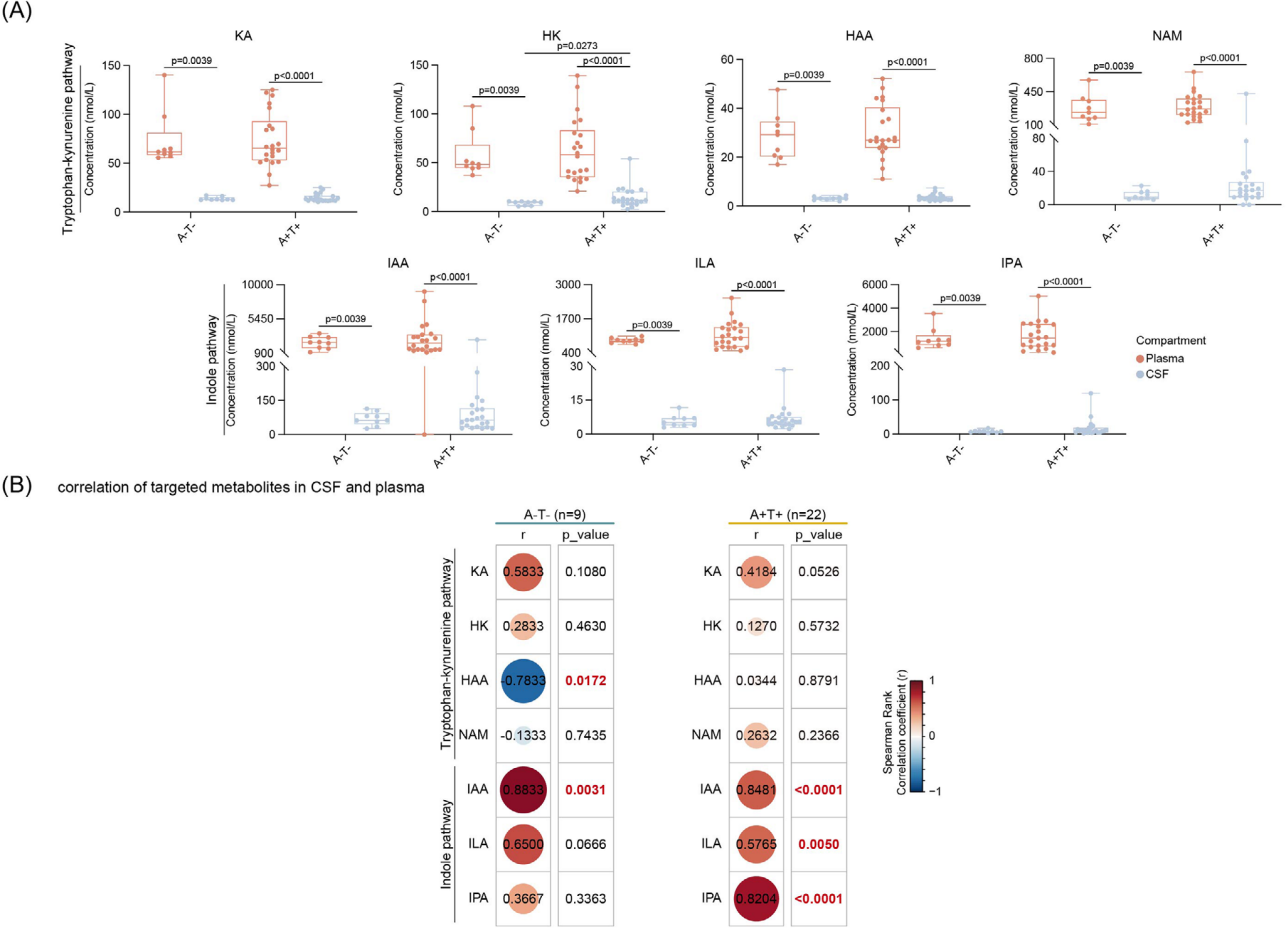
Cross‐compartment analysis of tryptophan metabolites in A‐T‐ and A+T+ individuals. (A) Box plots showing the concentrations of tryptophan metabolites in paired CSF and plasma samples from A‐T‐ and A+T+ groups. Statistical significance was assessed using the Wilcoxon matched pairs signed rank test. (B) Heatmap illustrating correlations between CSF and plasma concentrations of tryptophan metabolites in A‐T‐ and A+T+ groups. Correlations were determined using two‐sided nonparametric Spearman correlation tests (*r*). A, amyloid‐β; CSF, cerebrospinal fluid; T, tau.

Given that several discriminatory tryptophan metabolites (HK, HAA, NAM, IPA, IAA) identified in CSF may originate either from the gut–microbiota (indole pathway) or from endogenous synthesis in activated astrocytes or microglia (kynurenine pathway),^34^, ^35^ we next compared the levels of these metabolites in CSF and plasma (Figure 3(A)). Overall, all measured metabolites were present at higher concentrations in plasma as compared to CSF. This pattern supports a primarily peripheral origin for these metabolites, consistent with previous studies showing that kynurenine pathway activity occurs both systemically and centrally, but that peripheral kynurenine metabolites can cross the blood–brain barrier.^36^, ^37^ In CSF, both indole and kynurenine metabolites tended to be elevated in A+T+ patients, although HK, a direct metabolite of kynurenine, was the only metabolite significantly increased in A+T+ compared to A‐T‐ group (Figure 1(F), Figure 3(A)). Interestingly, NAM, a downstream metabolite of HAA and a precursor of NAD^+^, was detected at low levels in the CSF, but still higher than those of HAA. This pattern is consistent with reports that NAM can also be produced independently of the kynurenine pathway (e.g., via nicotinamide riboside salvage).^38^ Of the indole metabolites, IPA and its precursor ILA were found at lower levels in CSF. In contrast, IAA, which is a product of the indole‐pyruvate pathway‐was measurable and higher levels in both A‐T‐ and A+T+ individuals, suggesting differential CNS penetration or local production of specific indole derivatives.

We next examined the correlation between CSF and plasma metabolite levels across groups. In A‐T‐ individuals, HAA showed a significant negative correlation between CSF and plasma, whereas in A+T+ individuals, this relationship was lost. This pattern may reflect altered regulation of kynurenine catabolism in AD. Conversely, all measured indole metabolites (IAA, IPA, ILA) displayed significant positive correlations between CSF and plasma concentrations in A+T+ individuals only. Among A‐T‐ individuals, only IAA showed such a correlation (Figure 3(B)). These results suggest that while kynurenine metabolism (particularly HAA) may be disrupted in AD, indole pathway metabolites demonstrate stronger systemic‐to‐CNS coupling in the presence of AD pathology. Collectively, these findings suggest differential regulation and compartmentalization of tryptophan metabolism in AD. Specifically, altered kynurenine turnover (e.g., HAA) and enhanced peripheral‐to‐CNS coupling of indole metabolites may reflect disease‐associated changes in gut–brain communication, blood–brain barrier permeability, or metabolic clearance. Further studies are needed to determine whether these metabolite patterns contribute causally to AD progression or reflect compensatory responses.

**FIGURE 4:**
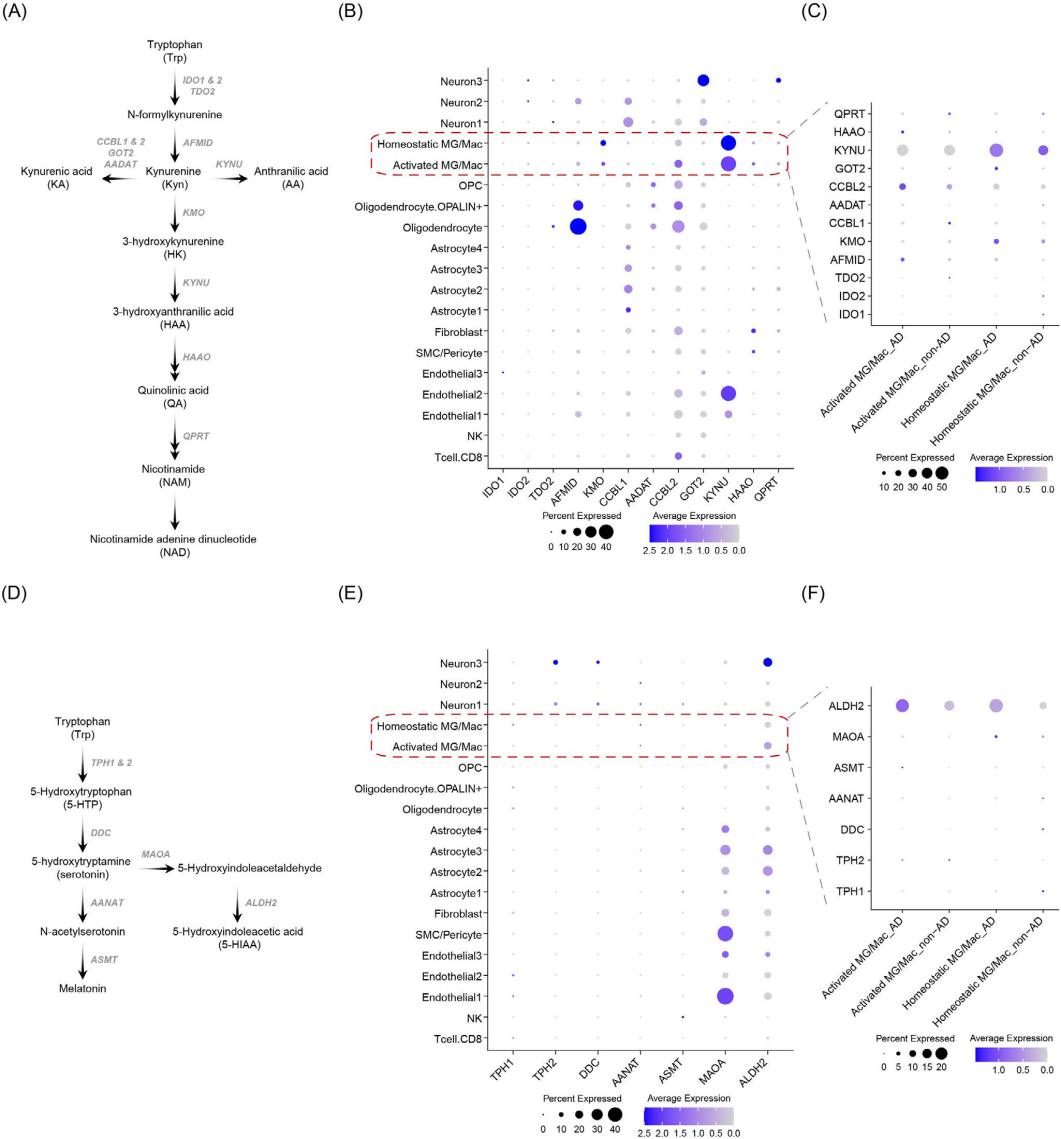
Expression of tryptophan pathway enzyme genes in *post mortem* brainstem samples from AD and non‐AD using Single‐nucleus RNA‐sequencing. (A) Scheme of tryptophan kynurenine metabolism. (B,C) Dot plots showing the expression of kynurenine pathway enzyme genes in brain cells (B) and MG/Mac (C) in AD (*n* = 4) and non‐AD (*n* = 8). (D) Scheme of tryptophan serotonin metabolism. (E,F) Dot plots showing the expression of serotonin pathway enzyme genes in brain cells (E) and MG/Mac (F) in AD (*n* = 4) and non‐AD (*n* = 8). Genes were considered significantly differentially expressed when they satisfied the criteria of log2(fold change) > 0.25 and *p* value < 0.05. *p*‐Values were adjusted using the Benjamini–Hochberg procedure, and significance was defined as an FDR‐adjusted *P* < 0.05. AD, Alzheimer's disease; FDR, false discovery rate; Mac, macrophage; MG, microglia.

As noted above, kynurenine pathway activity can occur both systemically and centrally. To investigate the potential for CNS tryptophan catabolism, we analyzed snRNA‐seq data from *post mortem* human brainstem samples of AD and non‐demented individuals.^28^ We first examined the expression of key enzymes involved in the kynurenine pathway (Figure 4(A)). Across all major brain cell types, microglia/macrophages (MG/Mac) showed predominant expression of KMO and KYNU, suggesting these cells as central mediators of kynurenine metabolism in the brain (Figure 4(B)). Notably, homeostatic MG/Mac exhibited higher KYNU expression than their activated counterparts, regardless of disease status, indicating a role in downstream kynurenine degradation. In contrast, activated MG/Mac from AD brains displayed elevated CCBL2 expression but lower KMO expression compared to homeostatic MG/Mac (Figure 4(C)), suggesting a shift toward increased kynurenine catabolism with enhanced KA synthesis and reduced HK production by activated MG/Mac. These findings imply that in AD, central HK may be predominantly derived from peripheral sources (Figure 3(A)). Conversely, homeostatic MG/Mac appear to favor kynurenine catabolism toward AA or HAA production but may have limited capacity to generate HK due to low *KMO* expression. We also evaluated the serotonin pathway (Figure 4(D)) and observed higher expression of ALDH2—an enzyme involved in serotonin catabolism—in activated MG/Mac, particularly in AD brains (Figures 4(E),(F)), pointing toward elevated central serotonin degradation in AD.

### 3.5 Cross‐compartment dysregulation of classical metabolites in AD

**FIGURE 5:**
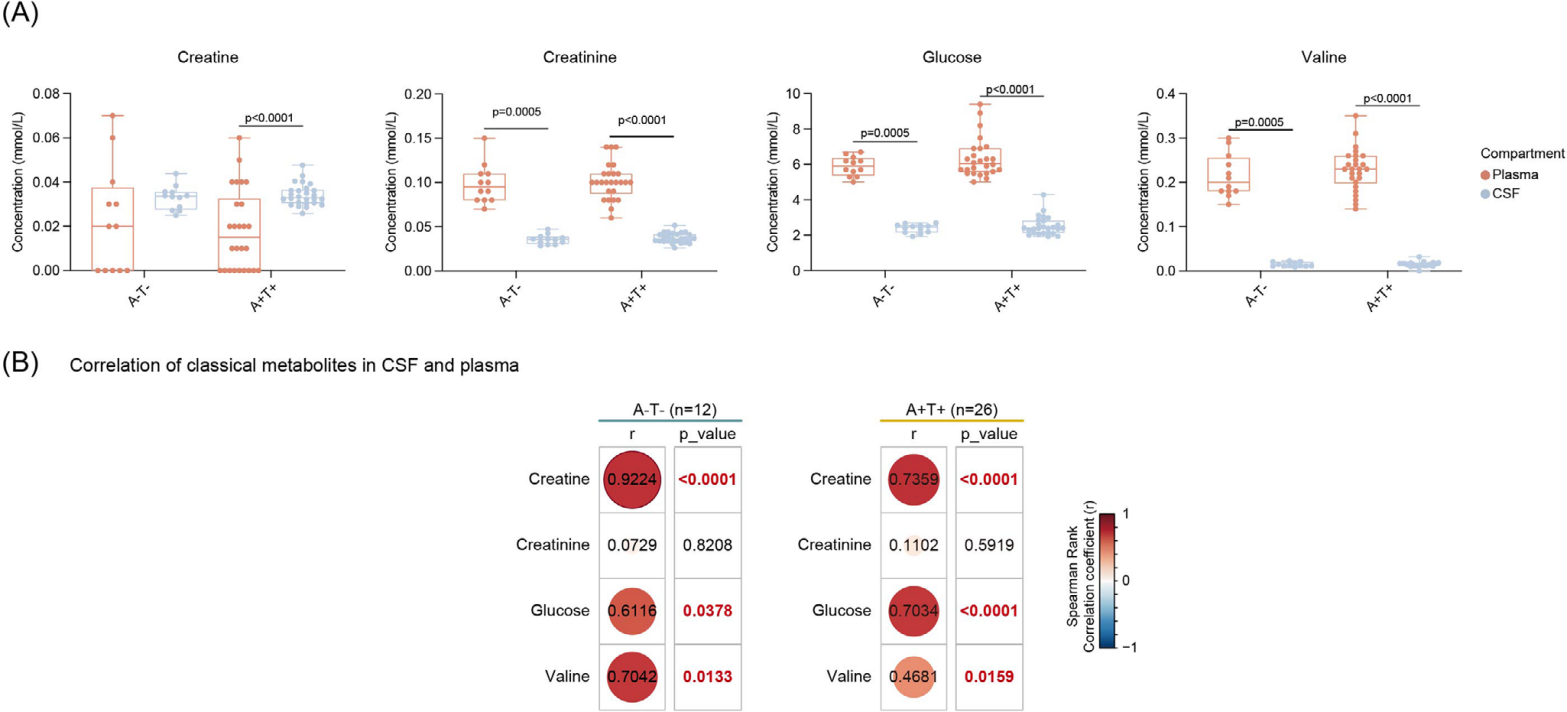
Cross‐compartment analysis of classical metabolites in A‐T‐ and A+T+ individuals. (A) Box plots showing the concentrations of classical metabolites in paired CSF and plasma samples from A‐T‐ and A+T+ groups. Statistical significance was assessed using the Wilcoxon matched pairs signed rank test. (B) Heatmap illustrating correlations between CSF and plasma concentrations of classical metabolites in A‐T‐ and A+T+ groups. Correlations were determined using two‐sided nonparametric Spearman correlation tests (*r*). A, amyloid‐β; CSF, cerebrospinal fluid; T, tau.

Beyond tryptophan‐derived metabolites, we quantified several classical metabolites involved in energy and BCAA metabolism in the CSF. Comparison of concentrations between CSF and plasma revealed that creatinine, glucose, and valine were consistently higher in plasma across both A‐T‐ and A+T+ groups. Interestingly, creatine, the precursor of creatinine, was present at significantly higher concentrations in CSF relative to plasma in A+T+ individuals, with a similar but non‐significant trend observed in A‐T‐ individuals (Figure 5(A)). Correlation analyses between CSF and plasma concentrations demonstrated that creatine, glucose, and valine were positively correlated across compartments in both groups, while creatinine levels were not consistently correlated (Figure 5(B)). These results reflect stable cross‐compartment distribution patterns of classical metabolites, without clear evidence of AD‐related dysregulation.

### 3.6 Blood‐based cellular and metabolic correlates of CSF proteomic changes in AD pathology

**FIGURE 6:**
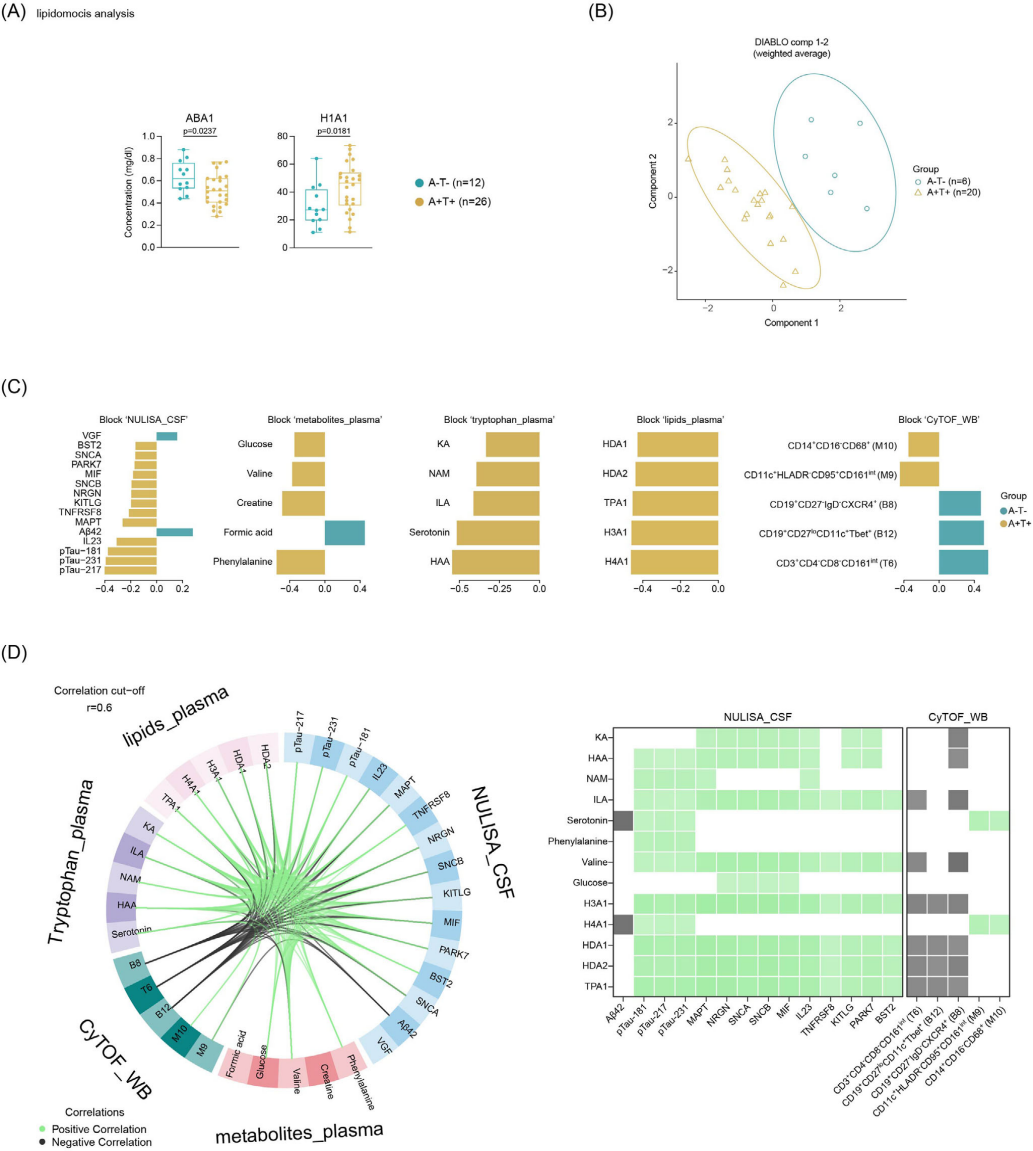
Multiple datasets integration of peripheral molecular signatures with CSF proteins distinguishes A‐T‐ and A+T+ groups. (A) Bar plots showing the concentrations of significantly altered lipoproteins in periphery between A‐T‐ and A+T+ groups. Statistical significance was assessed using the Mann–Whitney U test (two‐tailed, unpaired). To control for multiple testing, *p*‐values were adjusted using the two‐stage step‐up procedure of Benjamini, Krieger, and Yekutieli to control the false discovery rate at 5%. (B) Sample distribution in the two‐component space derived from supervised discriminant analysis using multiblock partial least squares (block PLSDA/DIABLO). (C) Loading plot of variables selected on Component 1. Features are ranked from bottom to top by decreasing absolute coefficient values and are colored according to the group (A‐T‐ or A+T+) with the higher median expression. (D) Circos plot displaying interaction between variables across omics blocks with absolute correlation coefficients ≥ 0.6. Green links represent positive correlations; black links represent negative correlations. Variables are positioned by block origin and colored accordingly. The right panel summarizes these correlations in matrix format, corresponding to the interactions shown in the Circos plot. A, amyloid‐β; T, tau; CSF, cerebrospinal fluid; DIABLO, discovery using latent components; sPLS‐DA, sparse partial least squares discriminant analysis.

Next, we investigated whether cellular and metabolite profiles in peripheral blood are correlated with CSF proteomic changes and whether these cross‐compartment association could reflect AD pathology‐related alterations observed in the CSF. Univariate statistical testing revealed a differential abundance of the ApoB100/ApoA1 ratio (ABA1), which was higher in A‐T‐ individuals, and high‐density lipoprotein (HDL) 1‐ApoA1 (H1A1), which was lower in A‐T‐ individuals (Figure 6(A)). No blood metabolites or immune cell subsets (Figure S8) showed statistically significant differences between the groups based on univariate analysis. Nevertheless, like our findings in CSF, integration of plasma metabolites (including lipoproteins) and immune cell profiles with CSF proteins using DIABLO revealed significant inter‐group differences (Figure 6(B,C). Consistent with findings in CSF, several tryptophan‐derived metabolites (HAA, serotonin, ILA, NAM, and KA) showed positive correlation with CSF DEPs and were positively associated with AD pathology. Interestingly, blood valine (the precursor of isobutyrate) was positively associated with AD pathology, whereas CSF isobutyrate exhibited an inverse relationship (Figure 2(B)). Moreover, phenylalanine, a diet‐derived amino acid and formic acid (a host‐derived one‐carbon metabolite) were both associated with AD pathology in opposing directions: phenylalanine was positively associated, while formic acid exhibited a negative association (Figure 6(C)). All discriminating lipoproteins, including Apo‐A1/2 associated HDL classes and subclasses (H3A1, H4A1, HDA1, HDA2) and total plasma Apo‐A1 (TPA1), were positively associated with A+T+ status, suggesting a potential link between apolipoprotein‐A1/A2 and AD pathology (Figure 6(C)). Similar to CSF immune cell alterations, we identified myeloid cell subsets in peripheral blood (CD14^+^CD16^−^CD68^+^ (M10) and CD11c^+^HLADR^−^CD95^+^ (M9)) that were positively associated with AD pathology. In contrast, lymphocyte subsets, including CD27^lo^CD11c^+^Tbet^+^ (B12), CXCR4^+^CD27^−^IgD^−^ (B8) B cells and CD161^int^CD4^−^CD8^−^ (T6) double negative (DN) T cells, were more prevalent in A‐T‐ individuals (Figure 6(C), Figure S3 for immune phenotype).

Next, we examined CSF‐plasma correlations in matched samples to assess cross‐compartment relationships between metabolites, lipoprotein (sub‐)classes, immune markers, and CSF proteins (|*r*| ≥ 0.6; Figure 6(D)). Tryptophan metabolites, including NAM, KA, HAA, and ILA, showed consistent associations across biofluids and were positively correlated with tau pathology (e.g., MAPT, pTau isoforms), immune markers (MIF, IL23), and neuronal proteins (NRGN, SNCB, SNCA). Similarly, plasma valine, mirrored the CSF correlation pattern of 2‐HIV, and was linked to tau and neuronal markers. In contrast, serotonin was only associated with Aβ42 (negatively), pTau isoforms (positively) and discriminating myeloid cell subsets (M10 and M9), hinting at a potential role in amyloid regulation and immune modulation. Correlation analysis revealed no significant associations between age and any of the metabolites examined (Figure S9), indicating that the observed group differences are unlikely to be age‐related. Notably, the Apo‐A1 associated HDL subclass‐H4A1 showed a similar pattern of associations.

Together, these findings reveal both conserved and compartment‐specific features: tryptophan and amino acid metabolites consistently tracked with tau and neuronal signatures across CSF and plasma, suggesting shared metabolic pathways in AD. However, immune correlations diverged (Treg and DCs dominated in CSF, while B and T cell subsets as well as myeloid cells were more relevant in plasma), highlighting compartment‐related immune responses in AD.

### 3.7 Correlations between MMSE scores and DIABLO‐identified metabolites in A‐T‐ and A+T+ individuals

Having established that several tryptophan‐related metabolites are differentially compartmentalized and associated with tau pathology, immune activation, and neuronal proteins, we investigated their potential relevance to cognitive function. Specifically, we assessed correlations between metabolite levels and MMSE scores in both CSF and plasma, stratified by AT status (A‐T‐ and A+T+ groups). Interestingly, in CSF of A+T+ individuals, KA, IAA, and ILA showed significant positive correlations with MMSE scores, suggesting that higher levels of these metabolites may be associated with better cognitive performance in the context of AD pathology (Figure S10(A)). These associations were not observed in the A‐T‐ group, indicating a potential disease relevance of these metabolites to cognitive outcomes. In contrast, in plasma of A‐T‐ individuals, MMSE scores were positively correlated with the apolipoprotein‐A1 associated HDL subclasses H3A1 and the apolipoprotein‐A2 associated HDL class HDA2, a relationship that was not significant in A+T+ individuals (Figure S10(B)).

## 4 DISCUSSION

In this study, we applied a comprehensive multi‐high‐dimensional integration strategy to paired CSF and blood specimens from individuals with and without AD pathology (A+T+ vs. A‐T‐). By integrating proteomic, metabolomic, lipidomic, and immune cell profiling from two different compartments, we identified both conserved and compartment‐specific cellular and molecular features associated with AD biomarkers. Tryptophan metabolism emerged as a consistent axis of immune–metabolic shift, linked to AD pathology and neuroinflammatory markers across compartments. While CSF immune signatures were dominated by regulatory T cells and DCs, peripheral blood associations involved myeloid as well as T and B cell subsets. Lipidomic and serotonergic alterations were also observed in the periphery. Together, these findings highlight coordinated yet compartmentally distinct immune–metabolic networks in AD and underscore the value of cross‐compartmental systems analysis for understanding disease mechanisms. Importantly, our study is exploratory in nature, aiming to map potential associations between biological systems (immune, proteomics, and metabolomics) and compartments (blood and CSF), rather than to establish causal relationships. As such, the observed cellular and molecular signatures should be interpreted as correlates, rather than direct contributors, to AD pathology. Our findings highlight the potential for the development of novel interventional or treatment strategies targeting multiple interconnected biological systems, with the goal of modulating immune–metabolic networks in AD.

Herein, we identified 13 DEPs in CSF associated with AD pathology, including established markers such as MAPT, pTau isoforms, Aβ42, and APOE4, alongside neuronal (VSNL1, SNCB, SNCA), metabolic (ENO2), and immune response proteins (MIF, IL23). Elevated MAPT and pTau are canonical features of tauopathy, while increased ENO2, a glycolytic enzyme enriched in neurons, reflects both metabolic dysregulation and neuronal injury in AD.^39^, ^40^ The pro‐inflammatory cytokine MIF, implicated in tau phosphorylation and Aβ toxicity, was also increased in A+T+ individuals, consistent with its role in neuroinflammation.^41^, ^42^ IL23 was significantly elevated in CSF in A+T+ individuals and correlated with other immune markers, representing a novel finding in biomarker‐defined AD cohorts. Previous studies have reported elevated IL23 in the serum of AD patients^43^ and increased levels of the p40 subunit, shared by IL12 and IL23, in the CSF,^44^ both of which are linked to heightened neuroinflammation and amyloid pathology. Experimental studies further demonstrate that targeting IL23 signaling, such as with anti‐p40 antibodies, can reduce amyloid plaque burden and improve cognitive performance in AD,^45^ supporting its potential role in disease progression.

Our integrative immune–proteomic analysis revealed divergent cellular associations across compartments. In CSF, CD1c^+^CD49d^+^ DCs and Tregs were associated with reduced tau pathology and inflammation, suggesting a protective immune environment in A‐T‐ individuals.^46^, ^47^ In contrast, CD1c^lo^CD49d^lo^ DCs and diminished Treg representation were linked to elevated IL23 and tau species in A+T+ individuals. These findings align with the known roles of DCs in immune surveillance and the protective effects of Tregs on microglial activation and Aβ clearance.^48^, ^49^

Tryptophan metabolism emerged as a consistent correlate of tau pathology across CSF and plasma. HK, a neurotoxic kynurenine pathway metabolite, was slightly elevated in CSF of A+T+ individuals. Both HK and its metabolite, NAM, showed positive correlation with pTau species and neuronal injury markers, suggesting the potential role of tryptophan–kynurenine metabolism in AD pathology. The snRNA‐seq data suggested increased kynurenine (Kyn) catabolism by *KYNU*‐expressing brain microglia/macrophage. Although these cells exhibited detectable expression of *KMO*, the enzyme converting Kyn to the neurotoxin HK, its expression level appeared insufficient to explain the elevated HK levels observed in AD CSF. This suggests that the accumulation of HK in the CSF may be predominantly driven by peripheral sources, rather than local CNS production.^50^ Although other tryptophan metabolites (HAA, NAM, IPA, IAA) showed no significant differences in univariate analyses, they exhibited notable correlations with proteomic and immune features in A+T+ individuals in multivariate integrative analysis. This suggests the involvement of the tryptophan‐kynurenine pathway in broader immune–metabolic networks relevant to AD pathology, even without overt groupwise concentration differences. Nevertheless, the alteration of kynurenine pathway has been also reported in other neurodegenerative diseases such as amyotrophic lateral sclerosis and Parkinson's disease.^51^ HAA, a downstream oxidative metabolite of HK, was positively correlated with MAPT and MIF. HAA's pro‐oxidant properties and association with reactive oxygen species (ROS) production suggest a potential role in neurodegenerative cascades.^52^, ^53^ NAM, a terminal kynurenine pathway metabolite and NAD^+^ precursor, also correlated with pTau proteins in both CSF and plasma. Its plasma levels exceeded CSF concentrations in both A+T+ and A‐T‐ groups, indicating predominantly peripheral production. While NAM is generally considered neuroprotective as a precursor for NAD+ biosynthesis,^54^ its elevation alongside tau pathology may reflect a compensatory response to neuronal injury or broader kynurenine pathway dysregulation in AD, especially in increased oxidative stress level conditions.^55^ KA, a neuroprotective metabolite with anti‐inflammatory properties, may mitigate neurodegeneration in AD.^56^, ^57^ In A+T+ individuals, plasma KA levels exceeded those in CSF, with a trend toward a positive inter‐compartmental correlation (*p* = 0.052). Consistently, activated microglia in AD showed increased expression of *CCBL2*, a key enzyme in KA synthesis, suggesting that both peripheral and central sources may contribute to KA levels in AD. As an NMDA receptor antagonist, KA is known to regulate synaptic plasticity,^56^ consistent with our finding that CSF KA levels positively correlated with MMSE scores in AD patients. Interestingly, an opposite trend was observed in A‐T‐ patients, suggesting a context‐dependent role of KA under AD‐related conditions. Additionally, plasma KA positively correlated with MAPT and MIF, further supporting a potential role for kynurenine pathway metabolites in response to AD‐related condition in the periphery.

Tryptophan‐indole pathway metabolites, including IAA, IPA, and ILA, are increasingly recognized as mediators of gut–brain communications.^58^ In our study, multivariate analyses revealed that CSF IPA and IAA correlated with AD‐related proteins (MAPT and FABP3) in A+T+ individuals. These findings suggest potential involvement of this alternative branch of tryptophan metabolism in AD and further support the contribution of gut‐derived metabolites to neurodegeneration.^59^, ^60^ These results are further supported by their positive correlations with MMSE scores. In plasma, ILA was positively associated with immune response proteins, including IL23 and KITLG. Given ILA's inhibitory effect on Th17 cell polarization,^61^ its positive correlation with IL23, a key regulator of Th17 differentiation, suggests that ILA may modulate Th17‐associated pathways. Interestingly, serotonin, another tryptophan‐derived metabolite, showed an inverse association with Aβ42 and CD11c^+^T‐bet^+^ B cells. CD11c^+^T‐bet^+^ B cells, a subset of age‐associated B cells,^62^ have recently been implicated in reducing inflammation and promoting amyloid clearance.^63^, ^64^

Beyond tryptophan metabolites, isobutyrate, a BCAA‐derived SCFA, was negatively correlated with CSF tau and neuronal proteins, suggesting neuroprotective potential.^65^ Prior studies have shown reduced isobutyrate levels in cognitively impaired individuals,^32^ and our data support its inverse relationship with AD pathology. Additionally, the valine derivative 2‐HIV was positively associated with MAPT and neuronal markers. Animal studies have demonstrated that BCAA supplementation (including valine) increases tau phosphorylation and cognitive impairment, whereas BCAA restriction delays cognitive decline and restores neurotransmitter levels.^66^ Furthermore, BCAAs may also impair tryptophan uptake into the brain and disrupt glucose metabolism.^67^ These metabolic disruptions may aggravate neuronal pathology, as impaired glucose metabolism‐ linked to neurodegeneration‐aligned with our observation that glucose levels inversely correlated with Aβ42. Collectively, these findings suggest that disrupted BCAA homeostasis contributes to AD progression, though causal mechanisms require further validation.

HDL lipid subclasses exhibited complex relationships with protein markers. ApoA1‐containing HDL subclasses (e.g., HDA1, HDA2, H3A1) were positively correlated with pTau and inflammatory proteins in plasma. ApoA1, the main HDL protein, has anti‐inflammatory and amyloid‐binding properties^68^, ^69^ and may cross the blood–brain barrier.^70^ Interestingly, the H4A1 subclass was uniquely negatively associated with Aβ42, suggesting that apo‐A1 associated HDL may influence amyloid clearance.^71^, ^72^, ^73^ These findings align with prior reports linking HDL subclasses to tau and Aβ metabolism and support further exploration of lipoprotein subtypes as biomarkers or therapeutic targets in AD.^74^

Cross‐compartment correlation analysis revealed shared metabolite‐protein relationships across plasma and CSF, particularly in A+T+ individuals. ILA and its downstream product IPA tracked closely between compartments, underscoring the permeability of microbial metabolites and their potential CNS influence in AD.^31^, ^75^ However, immune–proteomic correlations diverged significantly. CSF signatures featured DCs and Tregs, while blood was dominated by B and myeloid cell subsets. This compartment‐specific immune engagement likely reflects differences in cell trafficking, activation states, and local microenvironmental cues.

Taken together, our findings support a model wherein AD pathology involves coordinated metabolic, proteome, and immune alterations across CSF and peripheral compartments. This includes consistent tryptophan catabolism, disrupted BCAA and glucose metabolism, and lipid subclass‐specific associations with Aβ and tau. While sample size limits definitive conclusions, the robustness and biological plausibility of the multi‐omics associations lend support to these patterns. Our study also has technical limitations, including the focus on select immune cell panels and the absence of functional validation, as well as lack of a full panel of AD‐specific neurodegeneration (N) biomarkers. Future studies incorporating longitudinal data, broader omics coverage, and mechanistic validation will be crucial to advance these findings. In addition, further region‐specific and spatially resolved transcriptomic analyses are also required to refine understanding of CNS tryptophan metabolism in relation to AD pathology.

In conclusion, this study demonstrates the utility of integrative, cross‐compartmental high‐dimensional profiling in uncovering immune–proteome–metabolic features associated with AD. Tryptophan metabolites, especially those in the kynurenine pathways, emerged as consistent correlates of AD pathology. Compartment‐specific immune cell associations and lipoprotein subclass profiles further enhance our understanding of AD pathophysiology and its systemic consequences. These integrated signatures may inform the development of novel biomarkers and multi‐target therapeutic strategies. Future studies extending this approach to other neurodegenerative disorders and to anatomically distinct brain regions will be essential to determine whether these immune–metabolic signatures reflect conserved or disease‐specific mechanisms.

## CONFLICT OF INTEREST STATEMENT

The authors declare no conflicts of interest. Author disclosures are available in the Supporting Information.

## CONSENT STATEMENT

All human subjects provided informed consent.

## Supporting information



Supporting Information

Supporting Information
