# Supplementary material for: Immune–proteo–metabolomic changes link to Aβ and tau pathology in Alzheimer disease
Source: Alzheimers Dement. 2026 Apr 14;22(4):e71359. doi: 10.1002/alz.71359 (PMC13079071; doi:10.1002/alz.71359)
Supplement: Supplementary file 1 — Supporting Information [file ALZ-22-e71359-s002.docx]

# SUPPLEMENTARY MATERIALS

## SUPPLEMENTARY FIGURES


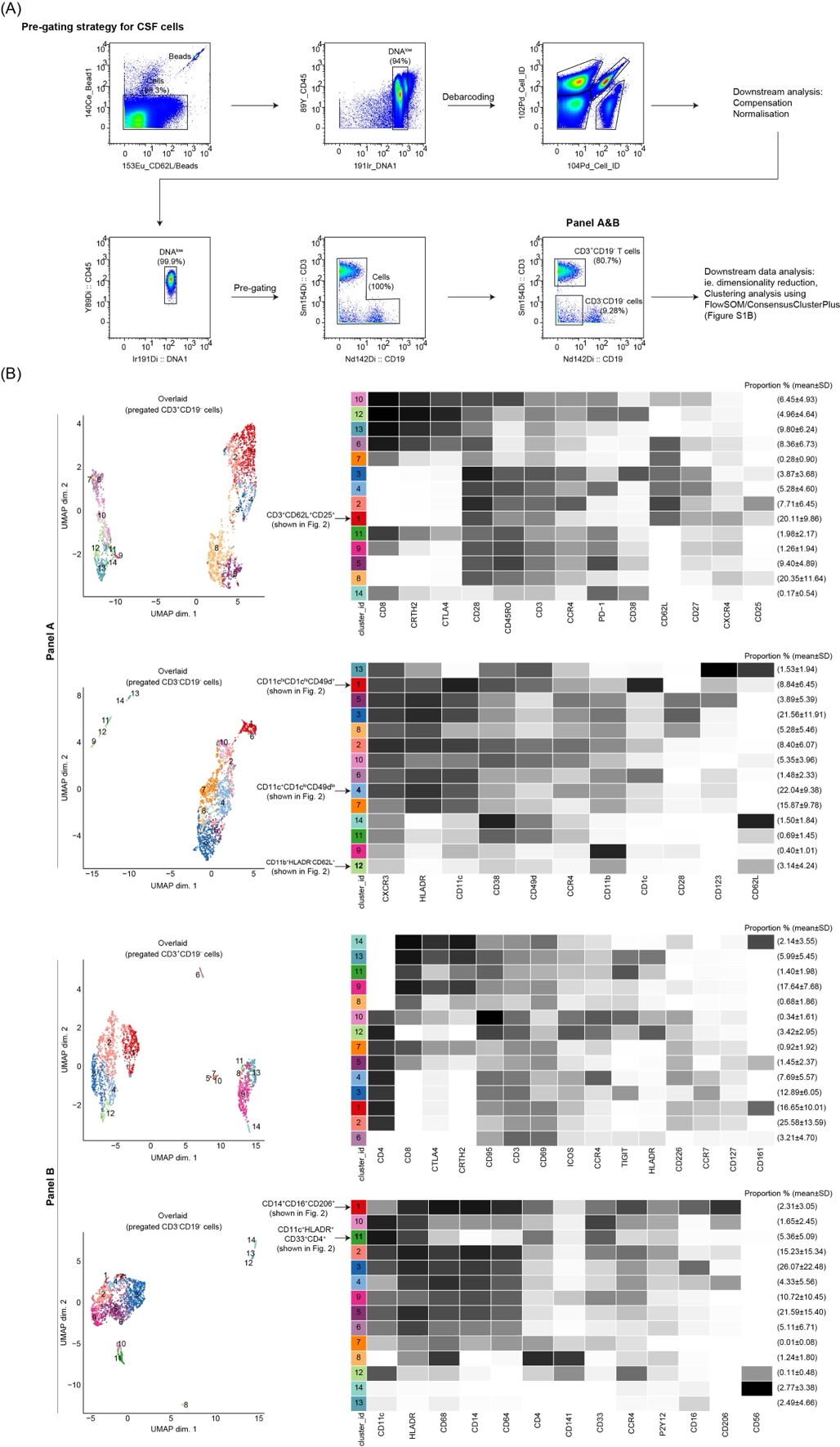


## Figure S1. CyTOF pre-gating strategy and unsupervised clustering of immune cell populations in CSF

(**A**) The mass cytometry (CyTOF) gating strategy includes sequential removal of cell debris, doublets, and beads, followed by de-barcoding. After these steps, the data files are then transferred to R for signal spillover compensation and batch normalization. Density plots in Panel A and Panel B display the gating strategies for CD3^+^CD19^-^ and CD3^-^CD19^-^ cell populations.

(**B**) Unsupervised clustering of CD3^+^CD19^-^ and CD3^-^CD19^-^ cells in Panel A and Panel B. Uniform manifold approximation and projection (UMAP) plots colored by cluster ID are shown on the left, with corresponding phenotypic heatmaps of median scaled expression presented on the right. Clusters highlighted in bold in the heatmap are those presented in Figure 2.

##
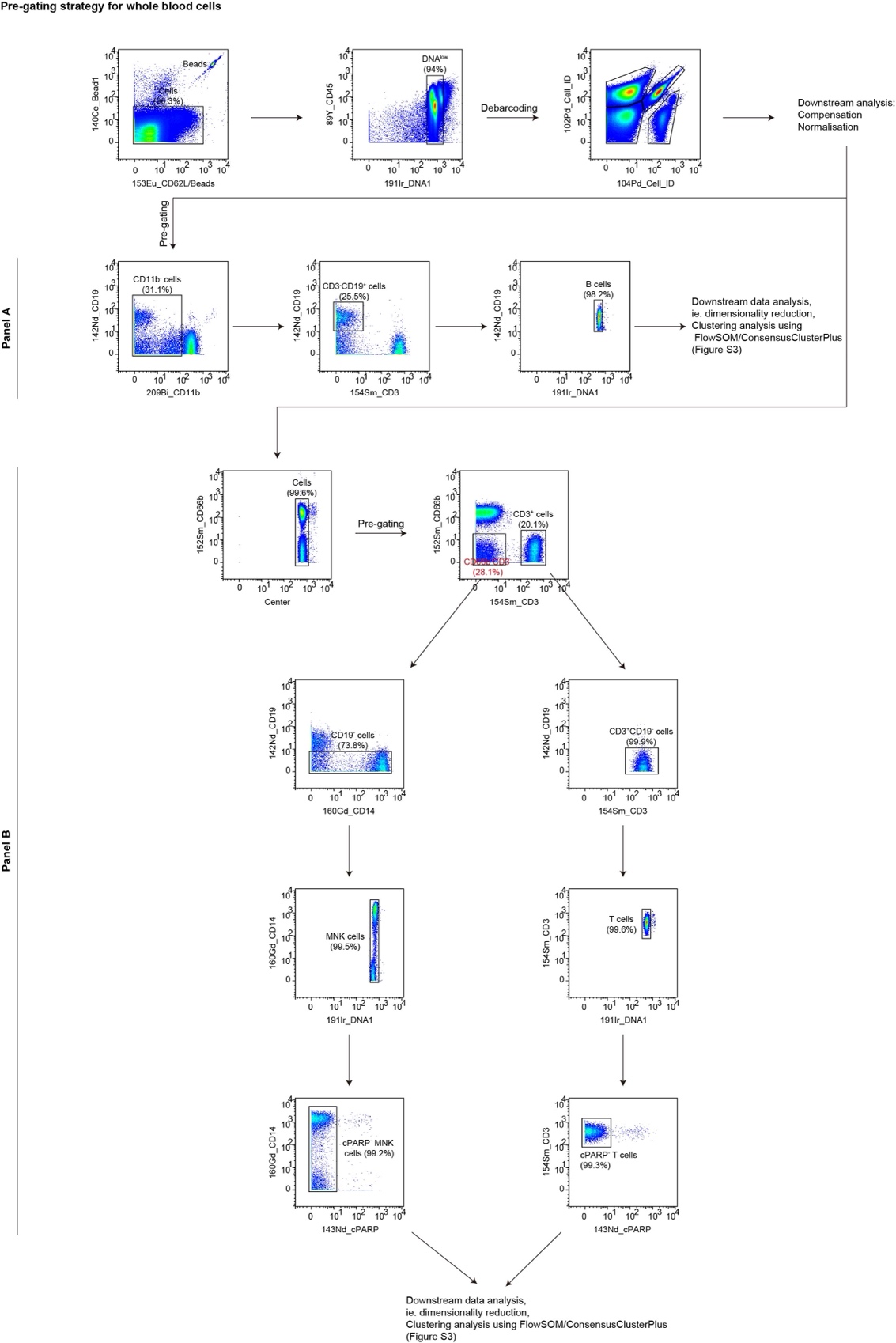


## Figure S2. Pre-gating strategy for whole blood cells

Gating strategy showing removal of cell debris, doublets and beads, followed by de-barcoding, after which files are transferred to R for compensation of signal spillover and normalization of signal intensity between batches. Gating strategies for B cells in Panel A, and myeloid and NK (MNK) and T cells in Panel B in density plots.


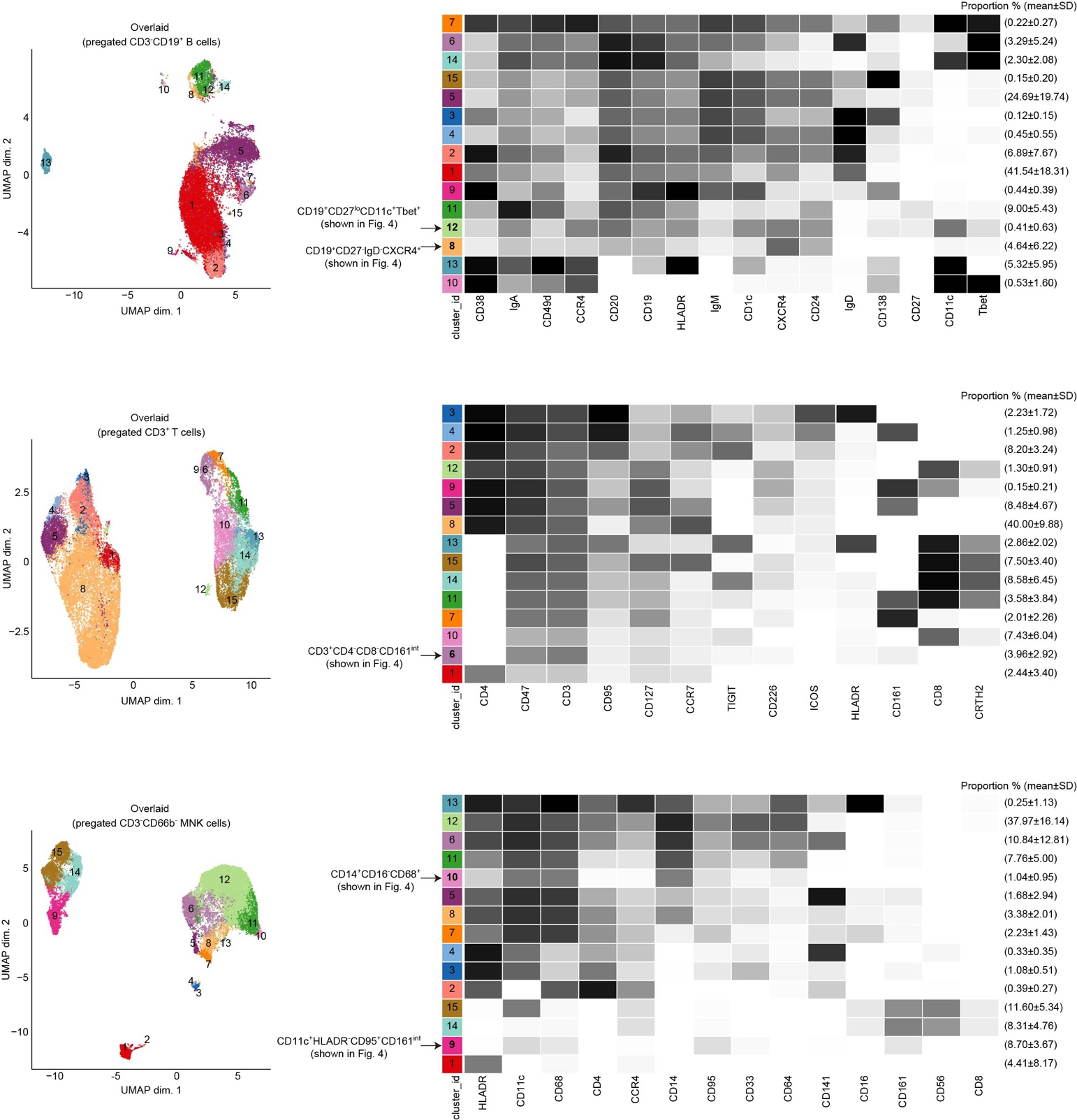


## Figure S3. Unsupervised clustering of immune cell populations in the peripheral blood

Unsupervised clustering of B cells (in Panel A), T cells and myeloid and NK (MNK) cells (in Panel B). UMAP plots colored by cluster ID are shown on the left, with corresponding phenotypic heatmaps of median scaled expression presented on the right. Clusters highlighted in bold in the heatmap are those presented in Figure 6.


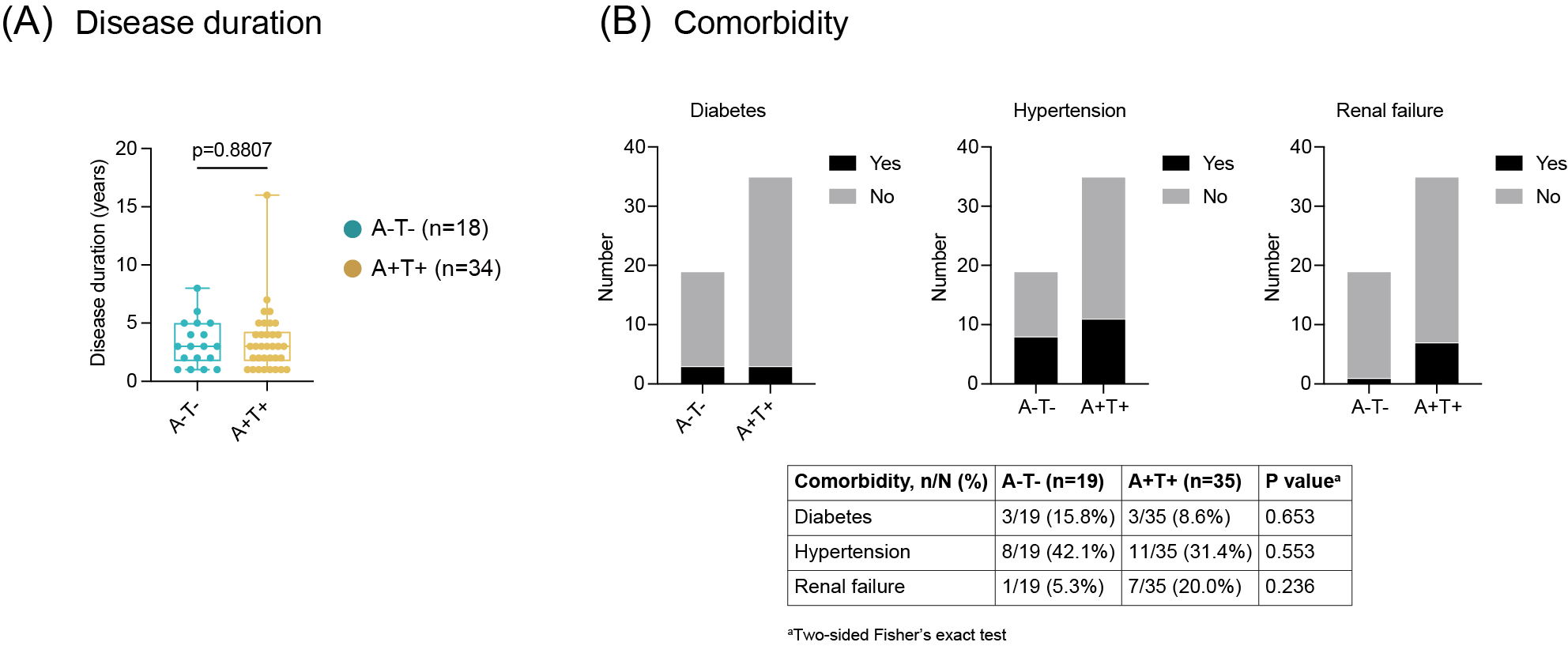


## Figure S4. Clinical information in A-T- and A+T+ groups

(**A**) Boxplots showing the disease duration (years) of A-T- (n=18) and A+T+ (n=34) groups. Each dot represents the value of each sample. Boxes extend from the 25th to 75th percentiles. Whisker plots show the min (smallest) and max (largest) values. The line in the box denotes the median. Statistical significance was determined using Mann–Whitney U-test.

(**B**) Stacked bar plots showing comorbidity frequencies (diabetes, hypertension, renal failure) in A−T− (n=19) and A+T+ (n=35). Bars show counts of Yes/No and are summarized as n/N (%). P values were calculated using two-sided Fisher’s exact test.


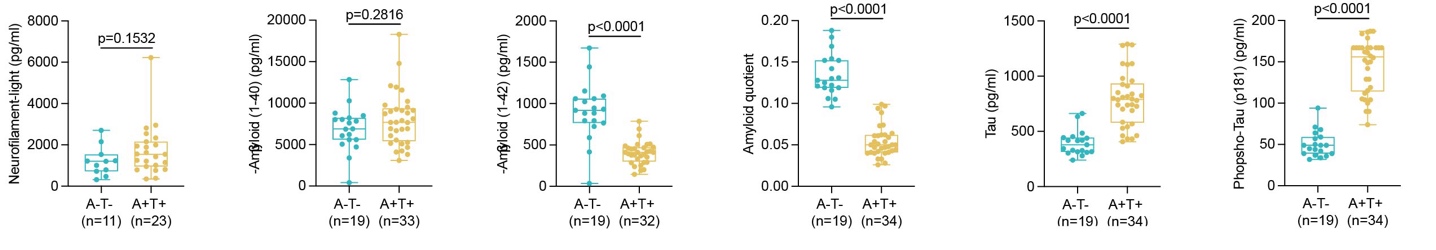


## Figure S5. Diagnostic biomarkers in A-T- and A+T+ groups

Boxplots showing the difference of diagnostic biomarkers between A-T- and A+T+ patients. Each dot represents one patient. Boxes extend from the 25th to 75th percentiles. Whisker show the min (smallest) and max (largest) values. The line in the box denotes the median. Statistical significance was determined using Mann–Whitney U-test. A, amyloid-β; T, tau. Amyloid quotient is Aβ(1-42)/Aβ(1-40) ratio.

**
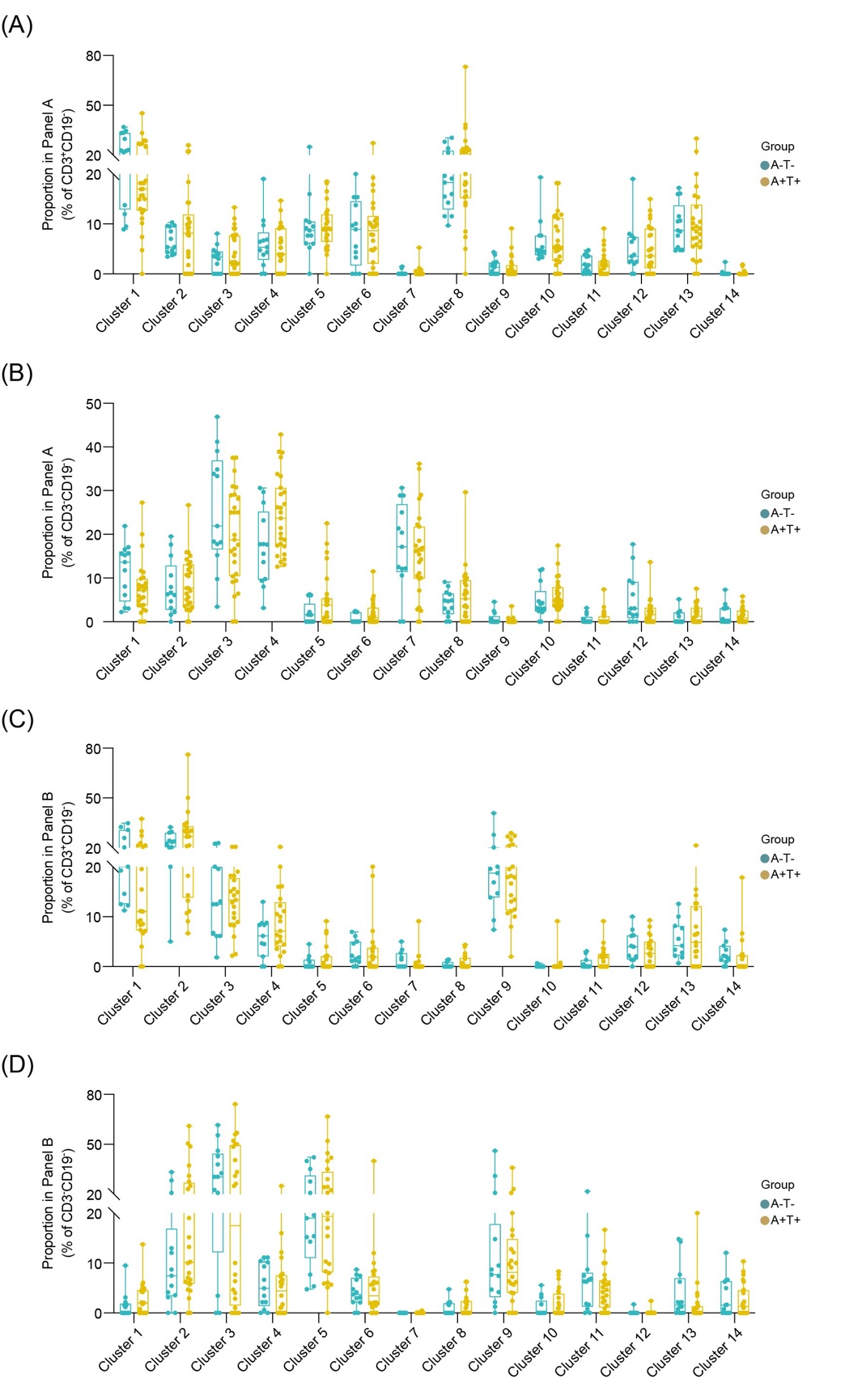
**

## Figure S6. Proportion of immune cells in CSF between A-T- and A+T+ patients

Boxplots showing the proportion of CD3^+^CD19^-^ cells (**A**) and CD3^-^CD19^-^ cells (**B**) in Panel A, as well as CD3^+^CD19^-^ cells (**C**) and CD3^-^CD19^-^ cells (**D**) in Panel B between A-T- and A+T+ patients. Each dot represents the value of each sample. Boxes extend from the 25th to 75th percentiles. Whisker plots show the min (smallest) and max (largest) values. The line in the box denotes the median.

**
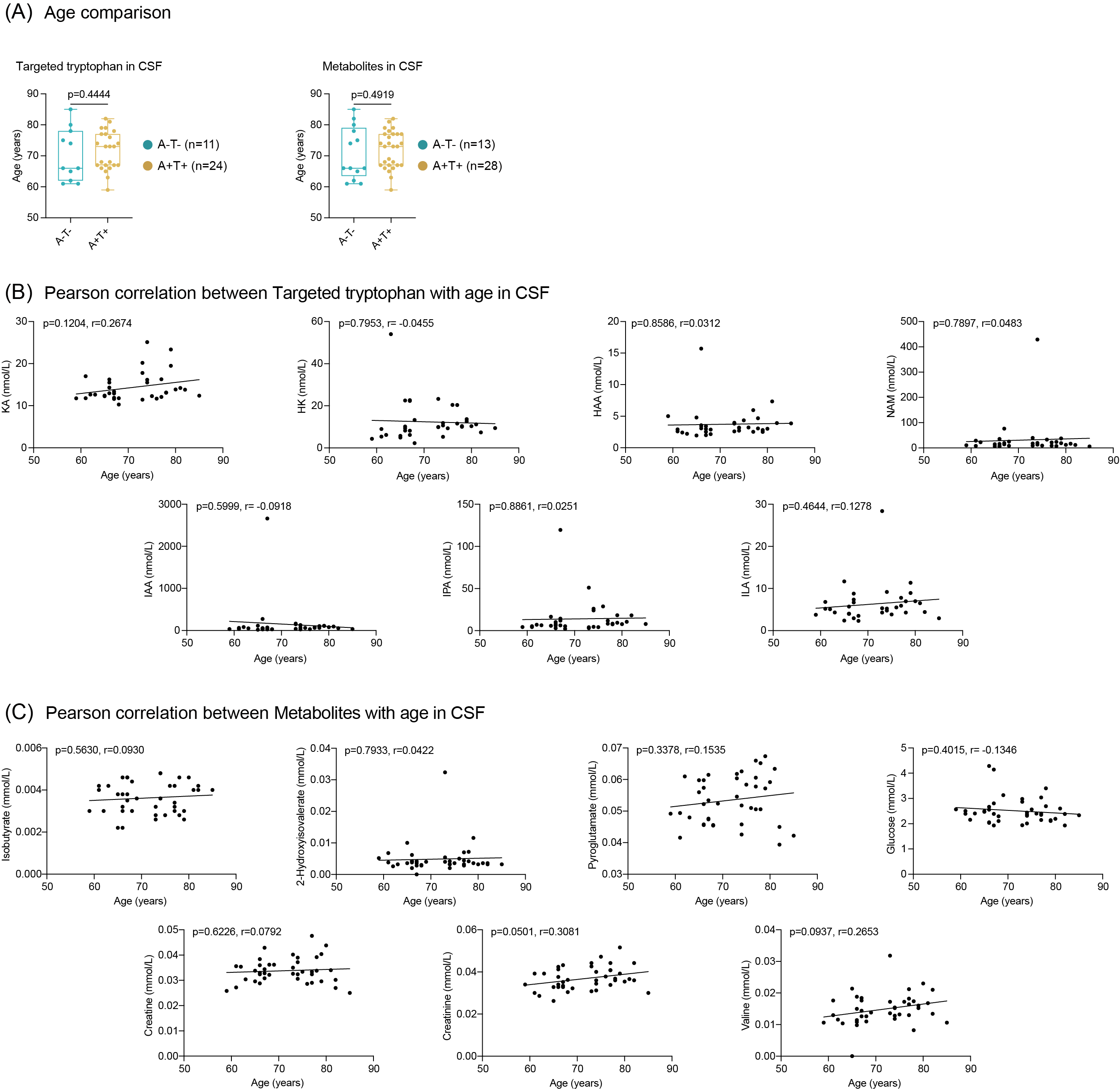
**

## Figure S7. Association between age and CSF metabolites

(**A**) Boxplots showing age (years) of A−T− and A+T+ participants included in the targeted tryptophan-metabolite dataset (left) and the metabolomics dataset (right) in CSF. Each dot represents one patient. Boxes extend from the 25th to 75th percentiles. Whisker show the min (smallest) and max (largest) values. The line in the box denotes the median. Statistical significance was determined using Mann–Whitney U-test.

(**B, C**) Scatter plots showing Pearson correlations between age and CSF targeted tryptophan metabolites (**B**) and CSF metabolites (**C**) (as in Figure 2) across all subjects. Each dot represents one sample; lines indicate linear regression fits. Pearson correlation coefficients (r) and two-sided P values are shown.


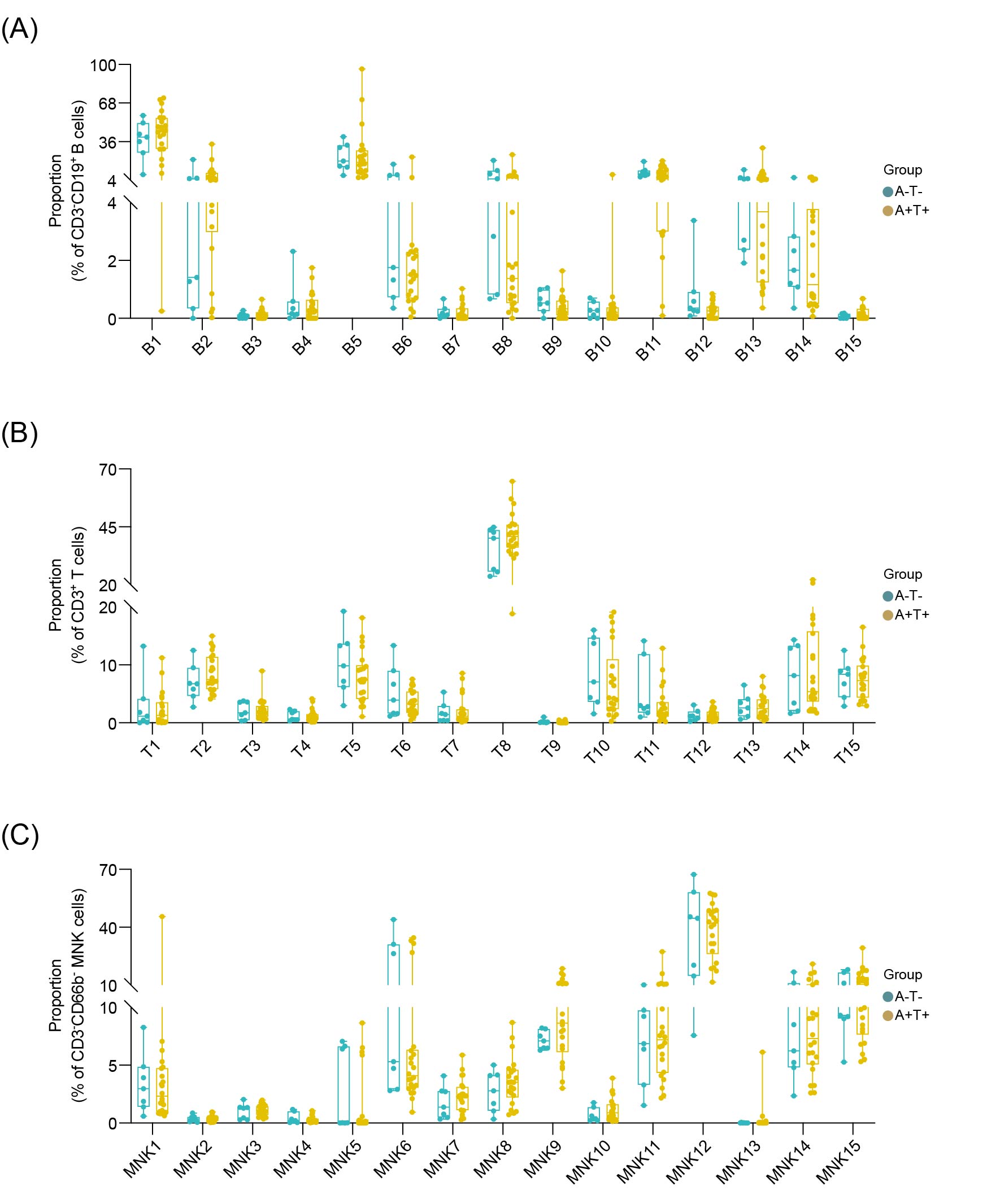


## Figure S8. Proportion of immune cells in whole blood between A-T- and A+T+ patients

Boxplots showing the proportion of CD3^-^CD19^+^ B cells (**A**), CD3^+^ T cells (**B**) and CD3^-^CD66b^-^ myeloid and NK (MNK) cells (**C**) between A-T- and A+T+ patients. Each dot represents the value of each sample. Boxes extend from the 25th to 75th percentiles. Whisker plots show the min (smallest) and max (largest) values. The line in the box denotes the median.

##
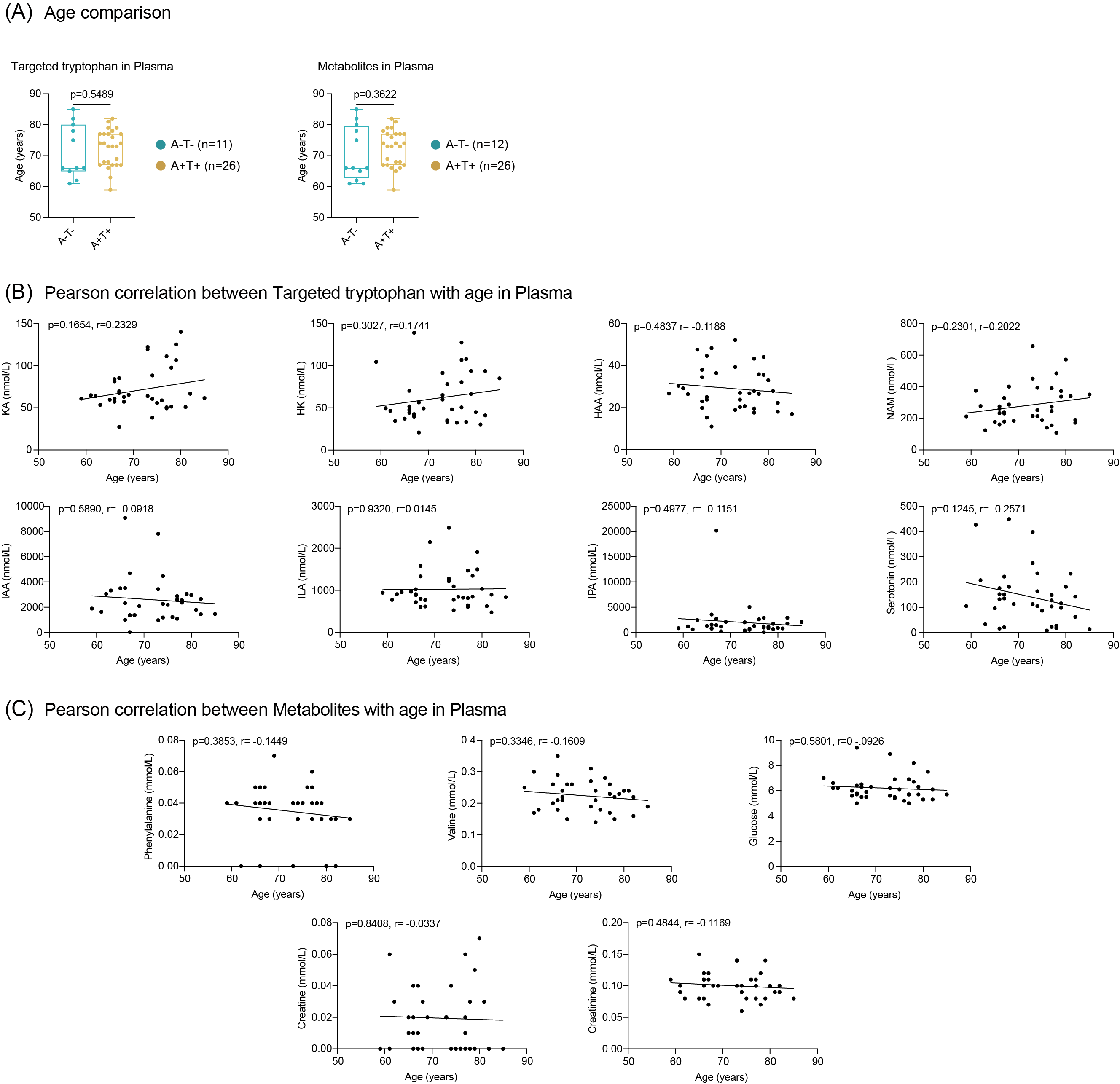


## Figure S9. Correlation between metabolites in plasma with age

(**A**) Boxplots showing age (years) of A−T− and A+T+ participants included in the targeted tryptophan-metabolite dataset (left) and the metabolomics dataset (right) in plasma. Each dot represents one patient. Boxes extend from the 25th to 75th percentiles. Whisker show the min (smallest) and max (largest) values. The line in the box denotes the median. Statistical significance was determined using Mann–Whitney U-test.

(**B, C**) Scatter plots showing Pearson correlations between age and plasma targeted tryptophan metabolites (**B**) and plasma metabolites (**C**) (as in Figure 6) across all subjects. Each dot represents one sample; lines indicate linear regression fits. Pearson correlation coefficients (r) and two-sided P values are shown.


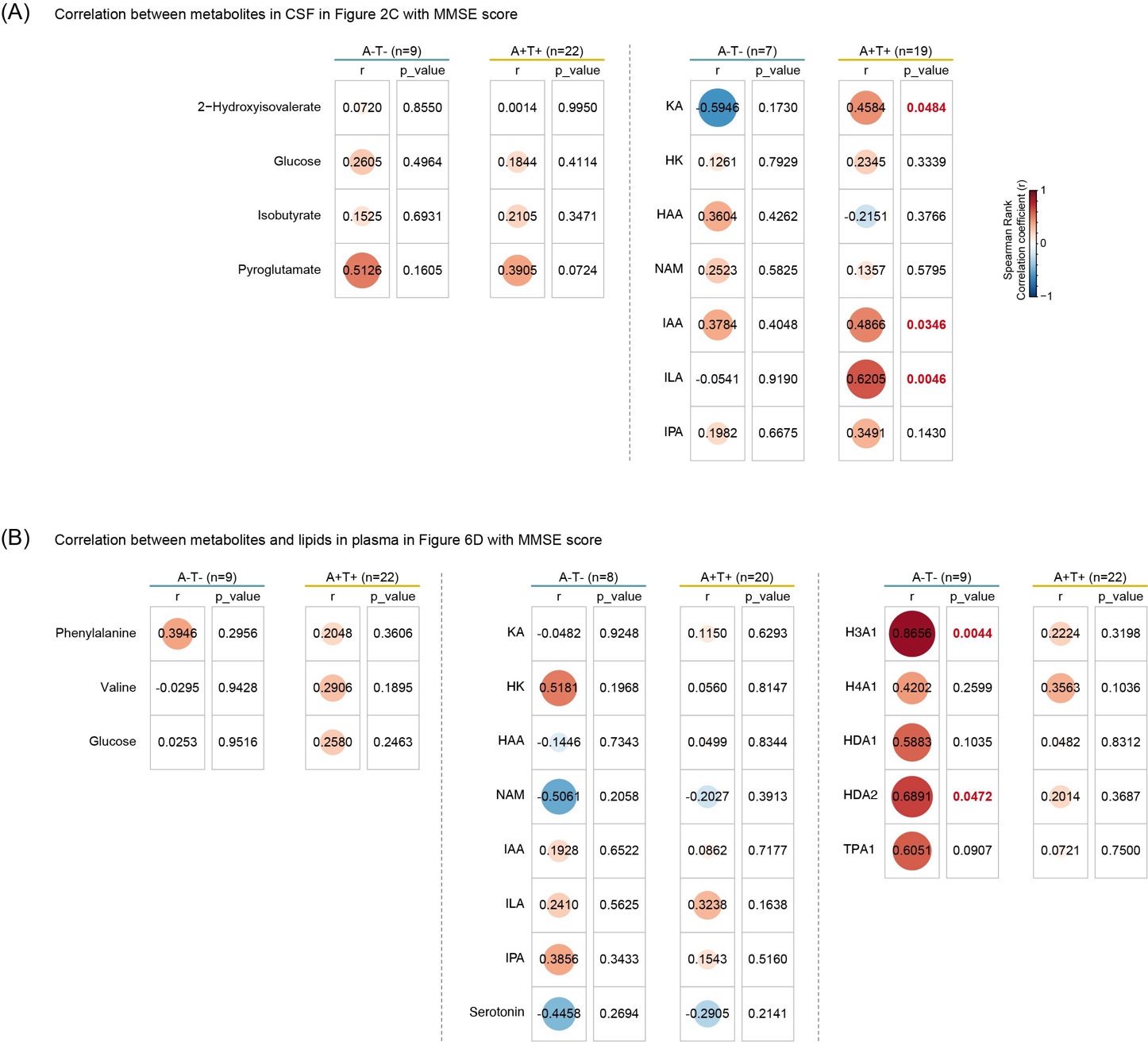


## Figure S10. Correlation of DIABLO-identified metabolites (in CSF and plasma) and plasma lipoproteins with MMSE score

(**A**) Heatmap of the correlation between metabolites in cerebrospinal fluid (CSF) (as shown in Figure 2) and Mini-Mental State Examination (MMSE) score. Nonparametric Spearman correlation test (r), two-sided. P-values shown in red bold font indicate statistical significance.

(**B**) Heatmap of the correlation between metabolites and lipoproteins in plasma (as shown in Figure 6) and MMSE score. Nonparametric Spearman correlation test (r), two-sided. P-values shown in red bold font indicate statistical significance.

# SUPPLEMENTARY TABLES

**Table S1. Clinical and demographic characteristics of each individual**

| **Sample_ID** | **AT catagory** | **Clinical diagnosis** | **Age** | **disease duration (years)** | **diabetes** | **high blood pressure** | **renal failure** |
| --- | --- | --- | --- | --- | --- | --- | --- |
| AT01 | A-T- | Cerebellar ataxia of unclear origin | 62 | 2 | no | no | no |
| AT02 | A-T- | parkinsonian syndrome | 75 | 2 | no | no | no |
| AT03 | A-T- | Behavioral-variant FTD | 66 | 3 | no | no | no |
| AT04 | A-T- | MCI | 82 | 5 | yes | yes | no |
| AT05 | A-T- | Corticobasal degeneration | 66 | 4 | no | no | no |
| AT06 | A-T- | Suspected temporal lobe epilepsy | 74 | 8 | no | no | no |
| AT07 | A-T- | MCI | 61 | 6 | no | yes | no |
| AT08 | A-T- | MCI | 85 | 1 | no | yes | no |
| AT09 | A-T- | Suspected neuroborreliosis | 66 | 5 | no | no | no |
| AT10 | A-T- | MCI | 61 | 4 | no | no | no |
| AT11 | A-T- | Somatic symptom disorder | 80 | 1 | no | no | no |
| AT12 | A-T- | SCD | 65 | 3 | yes | yes | yes |
| AT13 | A-T- | MCI | 78 | 2 | no | yes | no |
| AT14 | A-T- | Leukoencephalopathy of unknown etiology | 76 | 3 | no | no | no |
| AT15 | A-T- | MCI | 81 | 3 | no | no | no |
| AT16 | A-T- | MCI | 79 | 5 | yes | no | no |
| AT17 | A-T- | MCI | 63 | n.a. | no | yes | no |
| AT18 | A-T- | Depression | 64 | 1 | no | yes | no |
| AT19 | A-T- | Vascular dementia | 81 | 1 | no | yes | no |
| AT20 | A+T+ | EOAD (early onset Alzheimer disease) | 66 | 4 | no | yes | yes |
| AT21 | A+T+ | LOAD (late onset Alzheimer disease) | 65 | 4 | no | no | no |
| AT22 | A+T+ | LOAD | 79 | 3 | no | yes | no |
| AT23 | A+T+ | LOAD | 77 | 3 | no | no | no |
| AT24 | A+T+ | LOAD | 73 | 1 | no | no | yes |
| AT25 | A+T+ | LOAD | 78 | 2 | yes | yes | yes |
| AT26 | A+T+ | LOAD | 79 | 3 | no | no | yes |
| AT27 | A+T+ | LOAD | 73 | 1 | no | no | no |
| AT28 | A+T+ | LOAD | 81 | 1 | no | no | no |
| AT29 | A+T+ | LOAD | 77 | 7 | no | no | no |
| AT30 | A+T+ | LOAD | 68 | 5 | no | no | no |
| AT31 | A+T+ | EOAD | 59 | 3 | no | no | no |
| AT32 | A+T+ | LOAD | 66 | 2 | yes | yes | yes |
| AT33 | A+T+ | LOAD | 73 | 1 | no | no | no |
| AT34 | A+T+ | LOAD | 77 | 4 | no | yes | no |
| AT35 | A+T+ | LOAD | 74 | 5 | no | no | no |
| AT36 | A+T+ | LOAD | 69 | 2 | no | yes | no |
| AT37 | A+T+ | LOAD | 77 | 6 | no | no | no |
| AT38 | A+T+ | LOAD | 74 | 1 | no | no | no |
| AT39 | A+T+ | EOAD | 67 | 1 | no | no | no |
| AT40 | A+T+ | LOAD | 74 | 6 | no | yes | no |
| AT41 | A+T+ | EOAD | 68 | 5 | no | no | no |
| AT42 | A+T+ | LOAD | 67 | 3 | no | no | no |
| AT43 | A+T+ | LOAD | 82 | 2 | no | yes | yes |
| AT44 | A+T+ | LOAD | 76 | 5 | yes | yes | yes |
| AT45 | A+T+ | LOAD | 67 | 2 | no | no | no |
| **Table S1. Clinical and demographic characteristics of each individual (continued)** | | | | | | | |
| **Sample_ID** | **AT catagory** | **Clinical diagnosis** | **Age** | **disease duration (years)** | **diabetes** | **high blood pressure** | **renal failure** |
| AT46 | A+T+ | EOAD | 63 | 1 | no | no | no |
| AT47 | A+T+ | LOAD | 67 | 1 | no | no | no |
| AT48 | A+T+ | LOAD | 83 | 16 | no | no | no |
| AT49 | A+T+ | MCI | 77 | 2 | no | no | no |
| AT50 | A+T+ | MCI | 66 | not available | no | no | no |
| AT51 | A+T+ | MCI | 68 | 4 | no | yes | no |
| AT52 | A+T+ | LOAD | 70 | 4 | no | no | no |
| AT53 | A+T+ | LOAD | 54 | 3 | no | yes | no |
| AT54 | A+T+ | EOAD | 49 | 3 | no | no | no |

## Table S2. Antibody Panel A used for CyTOF measurements

| Panel A | | | | |
| --- | --- | --- | --- | --- |
| Target | Isotope tag | Clone | Company | Dilution |
| Surface | | | | |
| CD45 | ^89^Y | HI30 | Standard BioTools | 1:200 |
| CD49d | ^141^Pr | 9F10 | Standard BioTools | 1:100 |
| CD19 | ^142^Nd | HIB19 | Standard BioTools | 1:100 |
| HLA-DR | ^143^Nd | L243 | Standard BioTools | 1:200 |
| CD38 | ^144^Nd | HIT2 | Standard BioTools | 1:100 |
| CD138 | ^145^Nd | DL-101 | Standard BioTools | 1:200 |
| IgD | ^146^Nd | IA6-2 | Standard BioTools | 1:100 |
| CD123 | ^147^Sm | 6H6 | Biolegend | 1:100 |
| IgA | ^148^Nd | polyclonal | Standard BioTools | 1:200 |
| CD25 | ^149^Sm | 2A3 | Standard BioTools | 1:50 |
| KLRG1 | ^150^Nd | 14C2A07 | Biolegend | 1:100 |
| IgGL | ^151^Eu | MHL-38 | Standard BioTools | 1:200 |
| TCRgd | ^152^Sm | 11F2 | Standard BioTools | 1:100 |
| CD62L | ^153^Eu | DREG-56 | Standard BioTools | 1:100 |
| CD3 | ^154^Sm | UCHT1 | Standard BioTools | 1:200 |
| CD11c | ^155^Gd | Bu15 | Biolegend | 1:100 |
| CXCR3 | ^156^Gd | G025H7 | Standard BioTools | 1:200 |
| CCR4 | ^158^Gd | L291H4 | Standard BioTools | 1:200 |
| CD1c | ^159^Tb | L161 | Biolegend | 1:100 |
| IgGK | ^160^Gd | MHK-49 | Standard BioTools | 1:100 |
| CD8 | ^162^Dy | RPA-T8 | Standard BioTools | 1:50 |
| CRTH2 | ^163^Dy | BM16 | Standard BioTools | 1:100 |
| CD28 | ^164^Dy | L293 | BDBioscience | 1:50 |
| CD45RO | ^165^Ho | UCHL1 | Standard BioTools | 1:100 |
| CD34 | ^166^Er | 581 | Standard BioTools | 1:100 |
| CD27 | ^167^Er | O323 | Standard BioTools | 1:50 |
| CD24 | ^169^Tm | ML5 | Standard BioTools | 1:50 |
| Tbet | ^170^Er | 4B10 | Biolegend | 1:50 |
| CD20 | ^171^Yb | 2H7 | Standard BioTools | 1:100 |
| IgM | ^172^Yb | MHM-88 | Standard BioTools | 1:100 |
| CXCR4 | ^173^Yb | 12G5 | Standard BioTools | 1:200 |
| PD1 | ^174^Yb | EH12.2H7 | Standard BioTools | 1:50 |
| CD11b | ^209^Bi | ICRF44 | Standard BioTools | 1:200 |
| Intracellular | | | | |
| CTLA4 | ^161^Dy | 14D3 | Standard BioTools | 1:50 |
| Ki67 | ^168^Er | B56 | Standard BioTools | 1:50 |
| TNF | ^175^Lu | MAb11 | DVS Sciences | 1:50 |
| CHI3L1 | ^176^Yb | EPR19078-157 | abcam | 1:100 |

## Table S3. Antibody Panel B used for CyTOF measurements

| PanelB | | | | |
| --- | --- | --- | --- | --- |
| Target | Isotope tag | Clone | Company | Dilution |
| Surface | | | | |
| CD45 | ^89^Y | HI30 | Standard BioTools | 1:200 |
| HLA-DR | ^141^Pr | L243 | Biolegend | 1:100 |
| CD19 | ^142^Nd | HIB19 | Standard BioTools | 1:100 |
| CD69 | ^144^Nd | FN50 | Standard BioTools | 1:100 |
| CD4 | ^145^Nd | RPA-T4 | Standard BioTools | 1:100 |
| CD64 | ^146^Nd | 10,1 | Standard BioTools | 1:100 |
| CD226 | ^147^Sm | REA1040 | Miltenyi | 1:100 |
| CD16 | ^148^Nd | 3G8 | Standard BioTools | 1:50 |
| CD56 | ^149^Sm | NCAM16.2 | Standard BioTools | 1:400 |
| ICOS | ^151^Eu | C398.4A | Standard BioTools | 1:50 |
| CD66b | ^152^Sm | 80H3 | Standard BioTools | 1:600 |
| CD3 | ^154^Sm | UCHT1 | Standard BioTools | 1:200 |
| CD11c | ^155^Gd | But5 | Biolegend | 1:200 |
| CCR4 | ^158^Gd | L291H4 | Standard BioTools | 1:200 |
| TIGIT | ^159^Tb | MBSA43 | Standard BioTools | 1:50 |
| CD14 | ^160^Gd | RM052 | Standard BioTools | 1:100 |
| CD8 | ^162^Dy | RPA-T8 | Standard BioTools | 1:50 |
| CRTH2 | ^163^Dy | BM16 | Standard BioTools | 1:100 |
| CD95 | ^164^Dy | DX2 | Standard BioTools | 1:50 |
| LAG3 | ^165^Ho | 11C3C65 | Standard BioTools | 1:100 |
| CD141 | ^166^Er | M80 | Standard BioTools | 1:50 |
| CCR7 | ^167^Er | G043H7 | Standard BioTools | 1:100 |
| CD206 | ^168^Er | 15. Feb | Standard BioTools | 1:50 |
| CD33 | ^169^Tm | WM53 | Standard BioTools | 1:100 |
| FceR1a | ^170^Er | AER-37 (CRA1) | eBioscience | 1:100 |
| CD161 | ^171^Yb | HP-3G10 | Biolegend | 1:100 |
| CXCR4 | ^173^Yb | 12G5 | Standard BioTools | 1:200 |
| CD127 | ^176^Yb | A019D5 | DVS Sciences | 1:100 |
| CD47 | ^209^Bi | CC2C6 | Standard BioTools | 1:200 |
| Intracellular | | | | |
| cPARP | ^143^Nd | F21-852 | Standard BioTools | 1:200 |
| MIP1beta | ^150^Nd | D21-1351 | Standard BioTools | 1:100 |
| CD68 | ^153^Eu | Y1/82A | Biolegend | 1:100 |
| IL6 | ^156^Gd | MQ2-13AS | Standard BioTools | 1:50 |
| CTLA4 | ^161^Dy | 14D3 | Standard BioTools | 1:50 |
| OPN | ^172^Yb | polyclonal | LSBio | 1:50 |
| IL1beta | ^174^Yb | CRM56 | eBioscience | 1:50 |
| TNF | ^175^Lu | MAb11 | DVS Sciences | 1:50 |

## Table S4. Selected markers for clustering in CSF

| **Panel A** | |  | **Panel B** | |
| --- | --- | --- | --- | --- |
| **CD3^+^CD19^-^** | **CD3^-^CD19^-^** |  | **CD3^+^CD19^-^** | **CD3^-^CD19^-^** |
| CD3 | CXCR3 |  | CD3 | CD11c |
| CD8 | HLADR |  | CD4 | HLADR |
| CRTH2 | CD11c |  | CD8 | CD68 |
| CTLA4 | CD38 |  | CTLA4 | CD14 |
| CD28 | CD49d |  | CRTH2 | CD64 |
| CD45RO | CCR4 |  | CD95 | CD4 |
| CCR4 | CD11b |  | CD69 | CD141 |
| PD-1 | CD1c |  | ICOS | CD33 |
| CD38 | CD28 |  | CCR4 | CCR4 |
| CD62L | CD123 |  | TIGIT | P2Y12 |
| CD27 | CD62L |  | HLADR | CD16 |
| CXCR4 |  |  | CD226 | CD206 |
| CD25 |  |  | CCR7 | CD56 |
|  |  |  | CD127 |  |
|  |  |  | CD161 |  |

## Table S5. Selected markers for clustering in whole blood

| **Panel A** | **Panel B** | |
| --- | --- | --- |
| **B cells** | **T cells** | **MNK cells** |
| CD38 | CD3 | HLADR |
| IgA | CD4 | CD11c |
| CD49d | CD8 | CD68 |
| CCR4 | CD47 | CD4 |
| CD20 | CD95 | CD14 |
| CD19 | CD127 | CCR4 |
| HLADR | CCR7 | CD95 |
| IgM | TIGIT | CD33 |
| CD1c | CD226 | CD64 |
| CXCR4 | ICOS | CD141 |
| CD24 | HLADR | CD16 |
| IgD | CD161 | CD161 |
| CD138 | CRTH2 | CD56 |
| CD27 |  | CD8 |
| CD11c |  |  |
| Tbet |  |  |
